# Fungal and Microalgal Chitin: Structural Differences, Functional Properties, and Biomedical Applications

**DOI:** 10.3390/polym17202722

**Published:** 2025-10-10

**Authors:** Lijing Yin, Hang Li, Ronge Xing, Rongfeng Li, Kun Gao, Guantian Li, Song Liu

**Affiliations:** 1 Laboratory of Experimental Marine Biology, Institute of Oceanology, Chinese Academy of Sciences, Qingdao 266000, China; 2 Laboratory for Marine Biology and Biotechnology, Qingdao Marine Science and Technology Center, Qingdao 266000, China; 3 Laboratory for Marine Drugs and Bioproducts, Qingdao Marine Science and Technology Center, Qingdao 266000, China

**Keywords:** fungal chitin, microalgal chitin, chitosan

## Abstract

Chitin, one of the most abundant natural polysaccharides, has gained increasing attention for its structural diversity and potential in biomedicine, agriculture, food packaging, and advanced materials. Conventional chitin production from crustacean shell waste faces limitations, including seasonal availability, allergenic protein contamination, heavy metal residues, and environmentally harmful demineralization processes. Chitin from fungi and microalgae provides a sustainable and chemically versatile alternative. Fungal chitin, generally present in the α-polymorph, is embedded in a chitin–glucan–protein matrix that ensures high crystallinity, mechanical stability, and compatibility for biomedical applications. Microalgal β-chitin, particularly from diatoms, is secreted as high-aspect-ratio microrods and nanofibrils with parallel chain packing, providing enhanced reactivity and structural integrity that are highly attractive for functional materials. Recent progress in green extraction technologies, including enzymatic treatments, ionic liquids, and deep eutectic solvents, enables the recovery of chitin with reduced environmental burden while preserving its native morphology. By integrating sustainable sources with environmentally friendly processing methods, fungal and microalgal chitin offer unique structural polymorphs and tunable properties, positioning them as a promising alternative to crustacean-derived chitin.

## 1. Introduction

Chitin, a linear polysaccharide of β-(1→4)-linked N-acetyl-D-glucosamine, is the second most abundant biopolymer in nature after cellulose, widely distributed in arthropod exoskeletons, fungal cell walls, and microalgal frustules [1,2]. It has attracted considerable attention for its applications in biomedicine, agriculture, food preservation, and materials science, with functionalities ranging from drug delivery and wound healing to sustainable packaging [3,4,5].

Historically, commercial chitin was mainly extracted from crustacean shells [6]. However, this crustacean-based approach faces several significant limitations. Crustacean-derived chitin is subject to seasonal availability, contamination with allergenic proteins and heavy metals, and a high mineral content (30–60%) that necessitates harsh demineralization steps. These challenges increase both production cost and environmental impact [7]. These challenges limit scalability and clinical applicability, driving research into sustainable fungal and microalgal alternatives.

Fungal and microalgal sources have emerged as promising substitutes. Fungal chitin, particularly from edible and medicinal mushrooms such as *Agaricus bisporus*, *Lentinula edodes*, *Pleurotus ostreatus*, and *Ganoderma lucidum*, offers a renewable, hypoallergenic, and non-animal source of chitin [8]. Chitin content in mushrooms varies widely across species, ranging from 1.9% in *Lentinellus eoehleatus* to 44% in *Ganoderma lucidum* on a dry weight basis. Importantly, the mineral content of fungal chitin (2.5–7%) is substantially lower than that of crustaceans (30–60%), thereby reducing extraction complexity [9]. Cultivation of such fungi on agro-industrial residues enables year-round production, lower greenhouse gas emissions, and integration into the circular bioeconomy [10].

In addition, filamentous fungi and yeasts have been demonstrated as efficient microbial platforms for chitin and chitosan production. *Mucor rouxii*, for example, yields chitosan with a narrower molecular weight distribution and a higher degree of deacetylation, which enhances its biomedical suitability [11]. Similarly, the engineered yeast *Saccharomyces cerevisiae* has been used to produce high-quality α-chitin in scalable bioreactors, demonstrating the feasibility of controlled fungal platforms for chitin bioproduction [12]. The unique structural organization of fungal chitin (a chitin–glucan scaffold with glycoproteins) further contributes to its biological activity and functional versatility [13].

**Figure 1:**
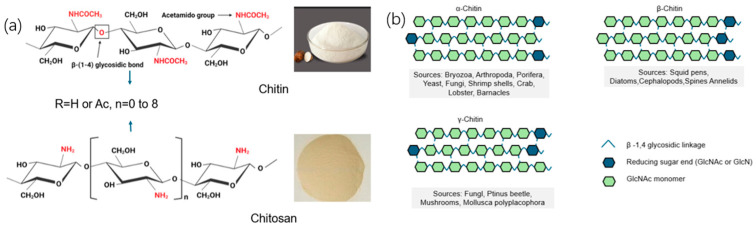
(**a**) Chemical structure of chitin and chitosan [18]. (**b**) Chitin has three allomorphs (α, β, γ), which differ in the orientation of the respective polymer chains within the micro-fibril macro structure [18]. Beyond industrial and biomedical contexts, fungal and microalgal chitin is also relevant as an ecological marker. For example, chitin content in arbuscular mycorrhizal fungi correlates with extraradical mycelial biomass, reflecting fungal activity in soil ecosystems [19]. This dual role as a structural biopolymer and as an indicator of ecological processes underscores its multifunctional significance.

Microalgal sources such as *Chlorella vulgaris* and diatoms (*Cyclotella* and *Thalassiosira*) offer additional benefits, with cell walls that integrate cellulose and chitin to provide enhanced mechanical stability [9]. These organisms grow rapidly without arable land, fix CO_2_ via photosynthesis, and lack lignin, which simplifies downstream processing [9]. Importantly, microalgal β-chitin exhibits distinct hydrogen-bonding patterns compared to crustacean α-chitin, providing greater reactivity and adaptability for chemical modification [14] (Figure 1). Studies further highlight that β-chitin from diatoms can be harvested as intact microrods without chemical degradation, expanding its promise in advanced biomedical and materials applications [15,16,17].

Recent research also emphasizes green extraction strategies, such as ionic liquids (ILs), deep eutectic solvents (DESs), and enzymatic hydrolysis, which minimize environmental burden and improve chitin purity [20,21]. These eco-friendly processes, combined with fungal and microalgal cultivation systems, position fungal and microalgal chitin as a sustainable, allergen-free alternative with strong translational potential across medicine, food technology, and industrial biotechnology [22,23,24].

In summary, fungal and microalgal chitin demonstrate structural richness, controlled purity, and functional versatility, positioning it as a superior alternative to crustacean-derived chitin. However, systematic comparisons of fungal and microalgal chitin regarding structural polymorphs, extraction methods, and structure–function relationships remain limited. This review synthesizes current advances in fungal and microalgal chitin research, addressing sources, preparation methods, bioactive properties, molecular mechanisms, and biomedical applications within the broader framework of the sustainable bioeconomy.

## 2. Sources and Preparation of Fungal and Microalgal Chitin

### 2.1. Fungal Chitin

Fungi provide a scalable, season-independent reservoir of chitin embedded within a chitin–β-glucan–protein cell wall matrices. Compared with crustacean sources, fungal feedstocks offer lower inorganic content, simpler demineralization, and valorization opportunities for agri-food side streams, while maintaining α-chitin allomorphy central to biomedical performance [25,26,27]. Historically, chitin was first isolated from mushrooms in the early 19th century, and progressive advances in purification and functionalization have enabled downstream production of chitosan and nanoscale derivatives tailored for biomedical use [26].

#### 2.1.1. Chitin from Mushrooms

Edible and medicinal mushrooms, particularly *Agaricus bisporus* and *Pleurotus* spp., are now recognized as practical, low-cost inputs because of their year-round availability, established cultivation infrastructure, and the appreciable chitin levels in caps and especially stipes [10,28]. Representative workflows start from fresh or dried fruiting bodies or from production residues/spent mushroom substrate (SMS). These materials undergo washing, size reduction, and thermal stabilization before sequential demineralization, deproteination, decolorization/bleaching, extensive washing, and drying/lyophilization. Typical chemistries include HCl (demineralization), NaOH (deproteination and, at higher concentration/temperature, deacetylation to chitosan), and optional oxidants/solvents for pigment removal; process intensification employs ionic liquids (ILs), deep eutectic solvents (DESs), enzymatic aids, and top-down mechanical defibrillation to access nano-objects.

**Figure 2:**
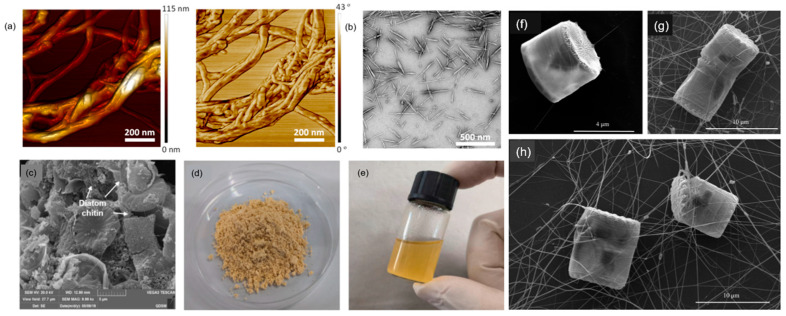
(**a**) Contact-mode AFM height and phase images of isolated ChNFs from *Agaricus bisporus*; (**b**) transmission electron microscopy image showing isolated ChNCs from commercial α-chitin powder under HCl treatment [33]; (**c**) SEM images of diatom; (**d**) diatom chitosan; and (**e**) diatom chitosan acetate solution [65]. SEM images of marine diatoms of genus *Thalasiosira* (**f**) and *Cyclotella* (**g**,**h**) shown with extracellular chitin nanofibers surrounding the cell. Whole cell and attached fibers [66].

In *Agaricus bisporus*, side-by-side comparisons show that classical alkaline pulping and imidazolium ILs can deliver high-purity chitin while preserving α-chitin crystallinity. For example, 1 M NaOH (24 h) or thermal treatment in [C2mim][OAc] achieved the highest purities (~77%), whereas lactic-acid: [Ch][Cl] DES maximized yield (30.6%) at a lower purity (~29.8%); intermediate routes included microwave-assisted [C2mim][OAc] (14.6% yield) and [C4mim][HSO4] pulping (23.8%) (Table 1) [29]. Industrially relevant SMS streams from *Agaricus bisporus* and *Pleurotus ostreatus* contain up to ~10% chitin (dry basis) and respond well to NaOH-based deproteination followed by mild acid washes, furnishing chitin suitable for bioconversion [30]. Pilot-scale handling of *A. bisporus* (washing, blending, 85 °C heat treatment, 1 M NaOH at 65 °C, repeated washing) yielded stable chitin/β-glucan dispersions; mechanical defibrillation produced chitin nanofibrils (ChNFs) at ~20.2 wt% on a dry basis (1.62 wt% of fresh biomass) [31]. Figure 2a,b show the isolation of ChNFs and chitin nanocrystals (ChNCs) from *Agaricus bisporus* and commercial α-chitin powder. Top-down routes routinely retain α-crystallinity and generate fibrils of a ~20–80 nm diameter and micron-scale length, aided by mild acid pretreatments and aqueous mechanical grinding [32,33,34]. Controlled HCl hydrolysis of purified *Agaricus bisporus* chitin (3 M, 120 min, 90 °C) yielded chitin nanocrystals with ~77% crystallinity, an average hydrodynamic size of 157 nm, and a high positive ζ-potential (+40 mV), achieving a recovery of ~76.8% [35]. Process knobs matter for mass balance and quality. In *Filamentous Ascomycota*, bleaching lowered chitin yield (42.1% unbleached vs. 8.2% bleached) but increased the relative chitosan recovery upon deacetylation (73.1% vs. 48.1 %), illustrating purity–yield trade-offs that should be tuned to target specifications [36]. Mild acetic acid protocols can replace harsh mineral acids to minimize protein/ash while preserving nanofibrillar morphology [10,37]. Enzymatic assistance (protease/β-glucanase) and hybrid enzyme–alkali schemes reduce chemical load while attaining high recoveries from *Agaricus bisporus* waste [38]. Alternative green solvents (NaOH/urea aqueous systems) maintain chitin–glucan integrity for fiber spinning and composite formation [13]. Across studies, yields and purities reflect species, tissue (stipe > pileus), pretreatment severity, and whether the goal is α-chitin, chitosan, ChNFs, or chitin nanocrystals [5,8,39,40,41,42,43,44]. 

#### 2.1.2. Chitin from Yeast

Yeasts accumulate comparatively lower chitin than filamentous fungi, and it is covalently integrated into a chitin–β-glucan complex (CGC); consequently, production strategies prioritize metabolic enrichment, CGC co-recovery, and gentle separations to preserve polymer integrity. Biotechnological enrichment in *Saccharomyces cerevisiae* via constitutively active CWI pathway alleles (e.g., RHO1^Q68H^ and PKC1^R398A^) increases wall chitin, allowing in situ quantification (Calcofluor White) and gravimetric validation without extensive deproteinization/demineralization [12]. High-throughput cell sorting of CW- or chitinase-probe-labeled populations yields mutant panels with elevated chitin suitable for fermentation-scale biomass recovery [52]. In *Pichia*/*Komagataella pastoris*, pathway engineering through overexpression of gfat, gna1, agm1, uap, and chs elevates wall chitin up to ~2.4-fold over wild type, demonstrating robust gene-stacking responses [46].

Downstream, hot alkaline extraction (∼1 M NaOH, ~65 °C) efficiently isolates yeast CGC with low protein/ash and characteristic β-glucan chitin ratios (e.g., ~75:25 mol) amenable to freeze-dried powders [53,54]. Process intensification benefits from waste-to-biomass platforms: *S. cerevisiae* on papaya waste and *Candida utilis* on potato juice waste generate cell walls rich in β-(1,3)/(1,6)-glucan with extractable chitin fractions; bead milling and protease polishing achieve >80% polysaccharide purity [47]. Adaptive evolution in *Rhodotorula toruloides* shifts wall carbohydrate composition and can be coupled with autolysis-assisted isolation and GC-based compositional control [55]. Developmental biology underpins timing: in filamentous model systems, CGC content peaks in sporophores and late-idiophase submerged mycelia, supporting harvest at stress-resilient stages for higher yields [56]. Collectively, yeast routes offer controllable sugar-to-polymer bioprocesses with flexible feedstocks and genetics, at the expense of lower absolute chitin fraction than mushroom stipes.

#### 2.1.3. Chitin from Other Fungi

*Filamentous Ascomycota* and *Zygomycota* furnish higher chitin/chitosan titers and diverse downstream options spanning cell wall chitin, partially deacetylated chitosan, nanofibers, and extracellular chitin-like polysaccharides. In *Fusarium incarnatum*, submerged cultivation on agro-industrial byproducts followed by alkaline purification (e.g., 1 M NaOH at sterilization temperatures) yields purified chitin suitable for biomedical upgrading [57,58]. Soil and marine isolates such as *Fusarium incarnatum* and *Penicillium chrysogenum* produce extractable chitin/chitosan via standard NaOH deproteination plus pigment quenching (KMnO_4_/oxalic acid), with reported chitosan degrees of deacetylation reaching ~94% [49,59]. Process optimization with response-surface methodologies in *Aspergillus terreus* increased chitosan titers nearly three-fold (to ~2.9 g L^−1^), illustrating the leverage of carbon/nitrogen balance, pH, and mineral ions [60].

Zygomycetes (e.g., *Rhizopus* and *Mucor*) are distinguished by chitosan-rich walls; solid-state NMR resolves multiple conformers of chitin and chitosan within rigid fractions, indicating polymorphic, highly cross-linked assemblies that influence extraction severity and product properties [61]. Practical routes include sequential alkaline purification, phosphate and chitosan removal, decolorization, and mechanical grinding to access chitin nanofibers (e.g., *Mucor indicus*) with preserved crystallinity [62]. Cost-effective nutrient strategies, such as replacing yeast extract with fungus-derived autolysates, support *Mucor rouxii* growth while maintaining or improving chitosan yields [48]. Co-production platforms integrate organic acid fermentations with chitin recovery (e.g., *Rhizopus oryzae* with lactic acid), stabilizing chitin content (~0.23–0.25 g glucosamine g^−1^ dry biomass) across batch and fed-batch regimes and improving economics on agricultural hydrolysates [63]. Beyond classic cell wall sources, submerged cultures of *Mortierella alpina* can secrete chitin-like extracellular polysaccharides separable by ethanol precipitation, expanding the accessible chemical space of fungal chitin analogs [8,64].

Across non-yeast fungi, the unifying extraction logic is as follows: (i) select strains/tissues with intrinsically higher chitin/chitosan content, (ii) tune media and stressors to promote wall fortification, and (iii) match purification severity (alkali/acid/enzymatic/green solvents) to the desired target—α-chitin, partially deacetylated chitosan, or nano-architected fibrils/crystals—while exploiting low-cost feedstocks and side-stream valorization to approach scalable, Q1-grade biomaterial specifications.

### 2.2. Microalgal Chitin

#### 2.2.1. Chitin from Diatoms

Diatoms are among the few microalgal groups that synthesize extracellular β-chitin, which is structurally distinct from the α-chitin found in fungi and crustaceans. The β-polymorph is characterized by parallel chain packing and an extensive hydrogen-bonding network that allows for reversible intercalation of polar molecules [14,22]. This unique molecular architecture underpins the mechanical flexibility and functionalization potential of diatom-derived chitin. In species such as *Thalassiosira rotula*, microrods of two distinct diameters are extruded through specialized frustule pores known as fultoportulae. These structures can be harvested using mild water-based methods involving centrifugation, salt-EDTA washes, and gentle shaking to detach the rods, followed by filtration and freeze-drying. Such approaches preserve the native ultrastructure of the β-chitin rods without resorting to harsh chemicals [15].

**Figure 3:**
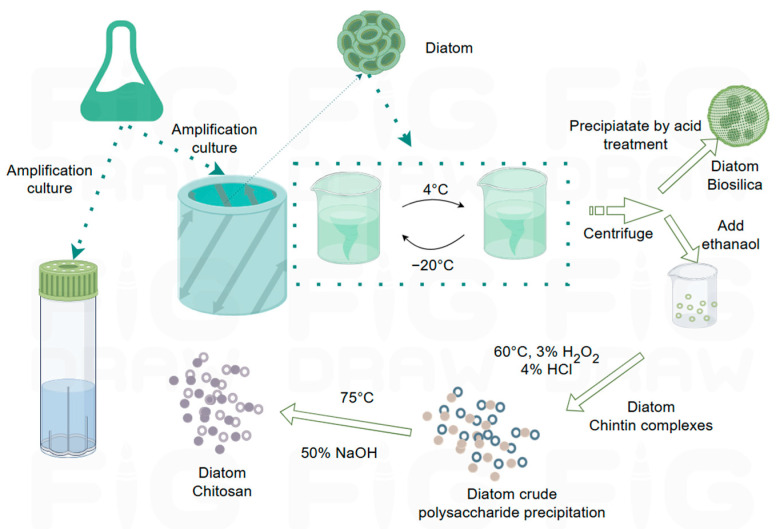
Schematic of the extraction of diatom chitosan (by Figdraw) [65].

Figure 2c–e feature diatom-derived chitin, specifically showing diatom chitosan and its isolated fibers. In *Cyclotella cryptica*, β-chitosan has been recovered using a urea–alkali freeze–thaw protocol followed by ethanol precipitation, depigmentation, and NaOH deacetylation, yielding homogeneous products comparable to crustacean-derived controls (Figure 3) [65]. Productivity varies widely across strains and conditions: *C. cryptica* isolates have achieved final chitin concentrations exceeding 300 mg/L, while *C. meneghiniana* produces an order of magnitude less [50]. Nutrient limitation plays a central role in modulating output. Silicon starvation strongly enhances nanofiber extrusion, with up to 79 fibers per cell reported under optimized conditions, whereas supplementation with trace germanium disrupts frustule morphology and reduces fiber number [67]. Phosphate and nitrogen delivery strategies also regulate co-production of lipids and chitin, with specific N/Si ratios maximizing yields [68,69]. Importantly, light intensity has little effect on chitin productivity, even though lipid output scales with irradiance, underscoring distinct regulatory pathways [70,71].

Beyond laboratory cultivation, environmental studies have confirmed diatom chitin production in situ. The freshwater diatom *Lindavia intermedia* produces mucilaginous threads that aggregate into “lake snow”, with Raman spectroscopy verifying these fibers as β-chitin [72]. Structural and functional investigations further highlight the advanced potential of diatom β-chitin. Pb^2+^ ions can be periodically aligned within the crystalline lattice of β-chitin succinate from *Thalassiosira weissflogii* [17], and strong interactions with silicic acid lead to silica–chitin nanocomposites [16]. Together, these findings illustrate that diatoms provide not only a sustainable microalgal source of β-chitin, but also a material with unique physicochemical properties highly suitable for biomedical and environmental applications.

#### 2.2.2. Chitin from Green Microalgae

Green microalgae represent a more complex and less consistent source of chitin, as the polymer is often a minor cell wall component whose abundance depends strongly on stress conditions or viral infection. *Chlorella vulgaris* has been the main model species in this context. Under nitrogen or phosphorus deprivation, or salinity stress ranging from 0.3 to 0.5 M NaCl, *Chlorella* cells exhibit thickened walls and up to threefold increases in biopolymer yield. The presence of chitin can be selectively identified by Wheat Germ Agglutinin (WGA) staining in combination with calcofluor white for cellulose, with quantification performed using flow cytometry and confocal microscopy [29].

Extraction protocols have emphasized wet biomass processing to avoid drying-induced cross-linking. A three-step green biorefinery approach has been proposed, as follows: solvent extraction of lipids and pigments, hot alkali treatment to remove proteins, and subsequent acetic/nitric acid purification to recover cellulose and a minor chitin fraction. This yields about 0.6% chitin on a dry weight basis while retaining material compatibility for downstream bioplastic production [9,51]. Viral infection also plays a notable role. Infections of *Chlorella* by chloroviruses have been shown to trigger extracellular chitin accumulation, with slow-growing isolates extending the accumulation window up to 12 h post-infection. Treatment with aphidicolin further enhanced yields by up to sixfold compared to standard infection, indicating that chitin biosynthesis is closely tied to cell cycle regulation [73].

Although the absolute yields from green microalgae remain relatively low compared to diatoms or fungi, these studies highlight two important directions. First, targeted environmental or biotic stresses can activate latent chitin biosynthetic pathways. Second, mild and integrated processing strategies ensure selective recovery of fragile polysaccharide fractions without damaging co-products. As such, *Chlorella* and related green microalgae should be considered promising, albeit conditional, fungal and microalgal sources of chitin, particularly in the context of circular biorefineries and sustainable biomaterial development.

## 3. Structural Differences in Fungal and Microalgal Chitin

Fungal and microalgal sources produce chitin with distinct molecular attributes that ultimately govern crystalline packing, supramolecular assembly, and macroscopic function. Here, we synthesize molecular-scale differences with a focus on the degree of polymerization (DP) and degree of acetylation (DA, or its complement degree of deacetylation, DD), presented in the following order: fungi (mushrooms, yeasts, and other fungi) → microalgae (diatoms and green microalgae). Figure 1 shows the chemical structures of chitin and chitosan, along with the following three chitin allomorphs: α, β, and γ. Panel (a) highlights the difference between chitin (acetylated) and chitosan (deacetylated). Panel (b) shows how the polymer chain orientations differ in the three allomorphs, with α-chitin being anti-parallel, β-chitin parallel, and γ-chitin having unique properties. These structural variations affect the material’s properties and are important for applications in fields like drug delivery and sustainable materials.

### 3.1. Molecular Structural Differences

#### 3.1.1. Degree of Polymerization (DP)

Top-down defibrillation of *Agaricus bisporus* produces chitin nanofibrils (ChNFs) with nanoscale diameters, demonstrating substantial chain scission relative to bulk cell wall chitin while preserving α-crystallinity [31]. Tissue-level surveys confirm that mushroom stipes are enriched in structural polysaccharide, consistent with longer native α-chitin chains before processing [28]. Mild mechanical routes that preserve fibrillar morphology similarly indicate extensive but controlled polymer breakdown during nanofibrillation rather than at the biosynthetic stage [33]. At the level of mushroom-derived chitooligosaccharides (COs), interspecies differences in bioactivity reported for *Pleurotus ostreatus*, *Cunninghamella bertholletiae*, and *Trichoderma viride* imply distinct precursor chain lengths and cleavage patterns during hydrolysis [74].

Engineering the cell wall integrity pathway in *Saccharomyces cerevisiae* (e.g., RHO1^Q68H^ and PKC1^R398A^) elevates wall chitin; fluorescence enrichment and mutant selection by FACS are consistent with longer and/or more abundant chains in situ, although absolute DP values were not reported [12,52]. In *Pichia*/*Komagataella pastoris*, pathway gene stacking increases chitin content up to 4.43-fold over wild type, compatible with enhanced polymer elongation during biosynthesis [46]. For the yeast CGC, regenerated *K. pastoris* polymers exhibit a weight-average molecular mass of Mw ≈ 4.9 × 10^5^ Da with PDI ≈ 1.7, consistent with a moderately high DP in a relatively narrow distribution for a fungal and microalgal copolymer [54]. Waste biomass yeast streams also preserve long β-glucan/chitin chains after alkaline extraction, indicating minimal depolymerization under gentle conditions [47].

Extracted chitin from *Fusarium incarnatum* retains long chains that support mechanical reinforcement roles in the wall [49]. Solid-state NMR of *Rhizopus delemar* and related Mucorales resolves multiple conformers within a rigid core composed almost entirely of long chitin/chitosan chains, an architecture indicative of high polymerization and dense cross-linking [61].

*Thalassiosira rotula* assembles chitin as microrods comprising bundled fibrils 5–30 µm in length (mean ~14 µm), an ultrastructure that implies a high DP despite the absence of direct chain-length measurements [15]. Species-dependent differences are evident: β-chitin nanofibers extruded by *Cyclotella cryptica* can reach 15–20 µm, substantially longer than those from *Cyclotella meneghiniana*, linking longer fibrils to higher polymerization potential and productivity [50]. Conversely, when diatom chitin is converted to chitosan, some systems yield relatively low and homogeneous molecular weights (e.g., *Cyclotella cryptica* chitosan with ~80% of species at ~50 kDa), underscoring controllable depolymerization during deacetylation/processing [65].

Cross-source comparison reveals that fungal α-chitin typically exhibits longer chains and tighter packing that reduce solubility, whereas diatom β-chitin is more reactive and amenable to intercalation/derivatization due to weaker interchain bonding and, frequently, lower effective DP after conversion to chitosan [14]. Within fungi, species and developmental stage modulate in-wall polymerization; within diatoms, species and extrusion morphology dominate fibril length scales [15,46,50,52].

#### 3.1.2. Degree of Acetylation (DA)

Multiple extraction routes for *A. bisporus* produce α-chitin with DA ~70–75% by NMR/FT-IR; thermal [C2mim][OAc] yielded ~75% DA, while 24 h NaOH produced ~72% [29]. SMS-derived materials show tissue-dependent differences after deacetylation: cap (Ch-C) and stalk (Ch-S) fractions converted to chitosan displayed markedly different residual acetylation (reported as DA ~97.1% for cap and ~82.3% for stalk prior to deacetylation of films), reflecting heterogeneous starting structures and processing responses [43]. Enzymatic or mild chemical extractions from mushroom waste can preserve higher acetylation than commercial α-chitin (e.g., DA 76.78–78.82% vs. ~75.05% in a benchmark material), consistent with gentler deproteinization (Table 2) [38]. Process severity strongly controls DA: 1 M NaOH maintains low deacetylation, whereas higher alkali (e.g., 8 M) substantially lowers DA in both store-bought *A. bisporus* powders and production residues [42]. During nanofibrillation, mushroom ChNFs generally retain the acetylation characteristic of fungal α-chitin [31]; covalently bound β-glucans can slightly depress the apparent DA (~75.8%) relative to crustacean α-chitin and alter surface chemistry/colloidal stability [33]. For *Pleurotus*, conversion to chitosan achieves application-relevant DD (∼72–81%), with bleaching and upstream chemistry shifting the DD/DA balance [36]. Historical and species-specific measurements corroborate α-chitin DA ≈ 70% in *A. bisporus* stipes and efficient deacetylation to chitosan without severe chain degradation in *Fomitopsis pinicola* (α-chitin DA 72.5%, chitosan DD 73.1%) [28,37,75,76]. Additional mushroom routes (e.g., TEMPO-oxidized *Filamentous Ascomycota* “ATC-Sponge”) selectively introduce C6-carboxyls and partially remove amorphous glucans, indirectly modulating the accessible acetylation landscape and glucosamine/glucose ratio [45]. Strong-alkali conversion of *A. bisporus* to chitosan (DD ~71.5%) yields values comparable to commercial analogs [39].

In *S. cerevisiae*, FT-IR confirms α-chitin acetylation patterns comparable to crustacean sources following genetic enrichment strategies [12]. Hot-alkali isolation of *Komagataella* CGC produces low-ash/protein material with native DA ~61%; dissolution–regeneration decreases DA to ~33.9–50.6%, evidencing partial conversion toward chitosan and highlighting process-induced acetylation drift [53,54]. Food-waste-derived yeast biomasses (e.g., *S. cerevisiae*, *Candida utilis*, *Pichia pastoris*, and *Candida robusta*) preserve acetylated α-chitin motifs within β-glucan–chitin complexes after purification, indicating that alkaline extraction can be tuned to minimize deacetylation [47]. In mutant-enriched *S. cerevisiae* populations, enhanced binding of GFP-tagged grape chitinases suggests greater exposure or stabilization of N-acetylglucosamine residues, which is consistent with higher local acetylation or increased chain accessibility [52].

Chitin from *Fusarium incarnatum* maintains acetylation within the α-chitin range prior to controlled deacetylation; processing can drive the resulting chitosan to DA ≈ 10% (i.e., DD ≈ 90%) akin to fishery sources [57,77]. *Fusarium incarnatum* isolates retain high DA by FT-IR, implying minimal acetyl loss during extraction [49]. In Zygomycetes, solid-state NMR reveals extensive deacetylation to chitosan with multiple allomorphs and dominant unprotonated amines stabilizing semi-crystalline domains—evidence of a regulated acetylation–deacetylation balance underpinning wall rigidity [61]. Broad fungal screens report DA spanning ~53.0–98.7%, with many species >80%, underscoring the capacity to obtain highly acetylated chitin without aggressive chemistry [10]. Reported DD values cluster around 69–75% for *Mucor rouxii* and ~71.9% for *Aspergillus terreus*, meeting typical solubility/bioperformance targets [48,60]. High-DD fungal chitosans (e.g., *Penicillium chrysogenum* DD ~ 94%) further illustrate the tunability of acetylation via fermentation and purification [59]. In composite extracts such as *Ganoderma lucidum* CGC, partial deacetylation (DDA ~ 46%) aligns with hemostatic performance in hydrogel formats [5]. Species-dependent DA/DD of COs (e.g., *T. viride* > *P. ostreatus* > *C. bertholletiae*) correlate with differential bioactivity profiles [74].

*Thalassiosira rotula* microrods are unequivocally β-chitin, implying high acetylation of the GlcNAc repeat; the β allomorph assignment indicates intact acetamide groups even without numerical DA [15]. Upon alkaline conversion, *Cyclotella cryptica* yields β-chitosan distinguished by FT-IR (amide I at 1628 cm^−1^), while crustacean α-chitosans show amide I near 1662/1624 cm^−1^; diatom chitosan also exhibits efficient deacetylation with narrow MW distributions [65]. Species-level extrusion of long β-chitin nanofibers (*C. cryptica* > *C. meneghiniana*) complements this picture by linking native acetylated β-chains to processable, reactive fibrils [50].

Fungal α-chitin typically presents higher DA with strong hydrogen-bonded packing, yielding a stable, less soluble polymer; diatom β-chitin combines high native acetylation with weaker packing, enabling facile deacetylation and derivatization [14,15,65]. Process parameters (alkali concentration, temperature, solvent system, and oxidative steps) systematically tune DA/DD across sources [29,42,53]. Genetic control (e.g., *Fusarium venenatum* chitin synthase knockouts) perturbs wall chitin abundance and, indirectly, the acetylation landscape, underscoring biosynthetic regulation as a lever on DA-linked properties [78].

**Table 2:** Summary of structural differences in fungal and microalgal chitin.

Source Type	Species	DP (Degree of Polymerization)	DA (%)	Chitin Polymorph	CI (%)	Morphology	Comparison with Crustacean Chitin	References
Mushroom	*Agaricus bisporus*	High (4.53 × 10^2^ kDa)	66–78.82	α	59–77	Fibers 20–80 nm wide, μm long	Lower DA, easier nanofibrillation	[20,29,38]
Mushroom	*Pleurotus ostreatus*	Medium (1.6 × 10^5^ Da)	31.7–80	α	58–73	Nanofibers ~28 nm wide	Lower CI, suitable for bioactivity	[43,79]
Yeast	*Komagataella pastoris*	Medium (4.9 × 10^5^ Da)	33.9–61.3	α	23–50	Dense fibril layers	Low DA, genetically tunable	[53,54]
Other Fungi	*Rhizopus delemar*	High	53–98.7	α/β	28–82	Polymorphic structure	Multiple polymorphs, easier deacetylation	[61]
Other Fungi	*Hericium erinaceus*	High (~337 nm long)	Not specified	α	49.2–70.8	Nanofibers 6.4 nm wide, 337 nm long	High aspect ratio, suitable for gels	[41]
Diatom	*Thalassiosira rotula*	High (5–30 µm long)	High	β	Not specified	Microrods 69–111 nm wide, 14 µm long	Open structure, reactive/insertable	[15]
Diatom	*Cyclotella cryptica*	High (15–20 µm long)	Not specified	β	Lower	Fibers 48–58 nm wide, 15–20 µm long	More soluble, suitable for hemostasis	[50,65]
Green Microalgae	*Chlorella vulgaris*	Low	Not specified	β	Not specified	Amorphous layered structure	Stress-induced, layered barrier	[51]

### 3.2. Crystalline Structural Differences

#### 3.2.1. Crystal Allomorphs

Across edible mushrooms, α-chitin is the prevailing polymorph. In *Agaricus bisporus*, FTIR (amide-I splitting) and ^13^C NMR (C3/C5 resolution) confirm α-type packing [20,29], consistent with the α-chitin signatures reported for *A. bisporus* fibers embedded in a glucan matrix [13], for α-chitin nanofibrils (ChNFs) retaining diagnostic XRD peaks at 2θ ≈ 9.2° and 19.7° with ~59% crystallinity [80], and for α-chitin nanocrystals (f-ChNCs) exhibiting canonical amide I/II bands (1624/1658/1560 cm^−1^) and α-type diffraction [35]. Mushroom- and shrimp-derived ChNCs, thus, share the α allomorph even though their mesoscale aspect ratios and liquid-crystalline self-assembly differ (helicoidal pitch ~360 nm for f-ChNC vs. ~4 µm for s-ChNC) [40]. Process history can, however, perturb polymorph: in *A. bisporus* powders, supercritical CO_2_ pretreatment induced β-like features relative to α-type obtained under mild extraction, implying stress-enabled polymorphic drift [42]. Inter-species and tissue contrasts accompany this chemistry: for cultivation residues, *A. bisporus* vs. *Pleurotus ostreatus* showed different DAs (~66% vs. 57%), with direct consequences for enzymatic digestibility and the hydrolysate COs profile, changes that often track subtle shifts in crystalline/amorphous partitioning even when the allomorph remains α [30]. Additional confirmations of α-type order appear in Irish *A. bisporus* wastes (FTIR/ssNMR) [38], in *Xanthoria parietina* and *Fomitopsis pinicola* [75,76], and in mixed chitin–glucan extracts where glucan dilutes crystallinity but not the α motif [37,81].

Regardless of metabolic engineering, *Pichia*/*Komagataella pastoris* chitin remains α-type, while engineered strains form thicker/denser fibril layers—i.e., changes in packing density rather than allomorph [46]. In *Saccharomyces cerevisiae*, XRD/FTIR similarly indicate α-chitin [12]. Yeast CGCs are semi-crystalline but retain α-type signatures after isolation or regeneration [53,54].

*Fusarium incarnatum* produces α-chitin, the dominant allomorph in filamentous fungi, with strong H-bonding and high stability [49]. *Zygomycetes* display unusual polymorphic richness: in *Rhizopus delemar* and relatives, solid-state spectra resolve four chitin (a–d) and four chitosan allomorphs; *R. delemar* expresses all major conformers, whereas some *Mucor* spp. lack type-c chitin and its associated chitin/chitosan–β-glucan complex, highlighting clade-specific polymorphism [61]. Chitin obtained via fermentation-assisted routes (e.g., *Flammulina velutipes* waste bioprocessed by *Fusarium incarnatum*) can present β-like features under certain chemistries [82], but most mushroom-derived nanofibrils/nanocrystals characterized to date remain α-type [31,35,80].

Centric diatoms predominantly extrude β-chitin. *Thalassiosira rotula* secretes β-chitin microrods through fultoportulae, featuring a more open H-bonding network that accommodates hydration and intercalation [15]. *Cyclotella cryptica* and *C. meneghiniana* generate β-chitin nanofibers [50], consistent with broader observations in *Cyclotella* spp. and “lake-snow” fibers of *Lindavia intermedia* and related centric taxa [67,70,71,72,83]. Upon deacetylation, diatom β-chitosan can partially convert toward α-type under acid–alkali protocols, whereas alkalization with freeze–thaw (urea–KOH) preserves β-type by shielding parallel sheets [65]. Cross-organism comparisons reinforce a mechanical dichotomy: β-chitin fibrils (e.g., *Loligo bleekeri* and *Lamellibrachia satsuma*) vs. α-chitin fibrils (e.g., *Phaeocystis globosa*) differ markedly in strength and deformation modes [84].

#### 3.2.2. Crystallinity Index (CI) and Thermal Stability

Processing route governs CI and thermal response in *A. bisporus*: 24 h NaOH afforded the highest CI (≈84.8%) and DTG_max ≈ 348.6 °C, whereas ionic liquids (ILs) and deep eutectic solvents (DESs) yielded a lower CI (≈63–66%) with reduced DTG_max (DES ≈ 300.9 °C) [29]. Cultivation residue studies show species-level contrasts: *A. bisporus* vs. *P. ostreatus* CI (≈63% vs. 73%), with higher crystallinity linked to lower enzyme accessibility and saccharification yields [30]. Chitosan films from *A. bisporus* caps vs. stalks (*P. ostreatus*) exhibit semi-crystalline XRD and two-step TGA (moisture ~6%, main degradation ~68%) with T_max ≈ 288 °C, confirming practical thermal stability [43]. Multiple works corroborate α-type XRD peaks and high stability relative to commercial comparators for mushroom wastes and for store-bought/residue *A. bisporus*, where moderate alkali preserves a higher CI than harsh alkali [42]. ChNFs from *A. bisporus* retain crystalline regions but, owing to glucan content and nanoscale size, show thermal stability up to ~280 °C and a CI of ~59% [31,33]. Fungal ChNCs often display sharper diffraction and earlier transitions to anisotropic phases than crustacean analogs due to their more homogeneous nanomorphology [40]. TEMPO-oxidized *Filamentous Ascomycota* “A-/ATC-Sponge” demonstrates that selective glucan removal and C6-oxidation shift the chitin/glucan ratio (32/68 → 64/36) and raise CI (≈47.1% → 51.0%), improving thermal/mechanical robustness [45]. Additional mushroom systems (e.g., *Hericium erinaceus* nanochitin) report CI increases (≈49.2% → 70.8%) with degradation peaks near 300–343 °C [41]. Typical chitin–glucan complexes from *A. bisporus* display a lower CI (≈54–64%) and degradation at 250–370 °C, reflecting amorphous glucan contributions [37].

Yeast CGCs are semi-crystalline with α-type order but a lower CI than crustaceans. In *Komagataella pastoris*, native CI can drop from ~35% to ~23–32% after dissolution/regeneration, with TGA onset shifting from ~302 °C to ~250–267 °C, consistent with partial conversion to chitosan–glucan complexes and disrupted crystallites [53]. Complementary measurements on pure CGC report CrI ≈ 50% and a main endothermic decomposition near 315 °C [54]. In *S. cerevisiae*, XRD/FTIR confirms crystalline α-chitin and thermal stability comparable to natural α-sources [12]. For *Penicillium*-derived chitosans (though filamentous, often discussed alongside yeast CGCs in downstream processing), a low CI and moderate stability are common, with major degradation between 175 and 320 °C [7].

Semi-crystalline α-chitin in *Fusarium incarnatum* correlates with stabilized biomass and yields over time [57]. *Fusarium incarnatum* chitin exhibits a high CI with TGA stability up to ~350 °C [49]. Composite films reinforced with fungal *Suillus*-derived ChNFs show higher crystallinity and elevated degradation onsets at 5–20 wt% loading [85]. In *Pleurotus ostreatus*, FTIR bands (e.g., 3340 cm^−1^ and 2937 cm^−1^) indicate a stable polysaccharide structure supportive of biomedical use [79]. Broader surveys report fungal chitin CI ≈ 28–78%, lower than crustacean α-chitin (≤~92%), with smaller crystallites (≈2.3–5.4 nm vs. 8–9 nm) and stability typically to ~240 °C [10]. For *P. ostreatus* ChGC, CI ~58–63% with DTG near 289 °C tracks higher amorphous glucan content and slightly diminished thermal stability relative to *A. bisporus* and marine α-chitin [81]. Classic medicinal fungi show robust α-order (e.g., *F. pinicola* DTG_max ≈ 341 °C; chitosan ≈ 265 °C) [75,76]. Growth/morphology also modulates alkali-insoluble material and GlcN/GlcNAc ratios in *Mucor rouxii*, reflecting adaptable crystallinity [48]. High-CI nanofibers (≈82%) are achievable from *Mucor indicus* [62]. In *Cunninghamella echinulata*, distinctive XRD peaks (2θ ≈ 13°, 9.5°) and a three-stage TGA (water ~50 °C; main 200–450 °C) with Tg ≈ 87.9 °C indicate stable semi-crystalline behavior [34]. Genetic perturbation (Δ*FvChs3*) expands hyphae and reduces wall electron density, consistent with altered crystalline organization [78]. FTIR in *Penicillium*-derived chitosan further confirms effective deacetylation with stable crystalline features [59].

Diatom chitosans exhibit a lower CI than shrimp/crab α-chitosan, consistent with the weaker intermolecular forces in β-type packing; TGA shows high purity (minimal ash) [65]. *Thalassiosira rotula* microrods display a tightly packed nanofibrillar architecture that withstands aqueous manipulation, indicating robust crystalline coherence despite the β-type openness [15]. For *Cyclotella* nanofibers, β-paracrystalline order is retained across strains [50,67,69].

#### 3.2.3. Crystallite Parameters

Scherrer analysis in *Agaricus bisporus* places crystallite sizes at ~3.8–4.9 nm for (020) and ~2.7–4.2 nm for (110), values smaller than typical marine α-chitin and consistent with mixed chitin–glucan walls [29]. Similar sub-10 nm crystallites have been cataloged across fungal sources [10]. Process-dependent refinement (e.g., TEMPO oxidation or nanofibrillation) can sharpen reflections and modestly increase apparent crystallite coherence by removing amorphous glucans [41,45].

In *K. pastoris* CGCs, dissolution/regeneration narrows or broadens lattice coherence depending on fractionation, tracking CI decreases [53,54]. Semi-crystalline halos without the sharp (020) chitin reflection typify Penicillium chitosans [7], reflecting a lower long-range order than crustacean α-chitin.

*Fusarium incarnatum* from Egyptian soils exhibits α-chitin lattice parameters characteristic of well-ordered crystallites [49]. In Mucorales, multiple chitin/chitosan conformers imply a family of crystallite microenvironments rather than a single lattice state [61].

Hierarchical assembly is prominent: *Thalassiosira rotula* builds microrods from bundled nanorods ~16–20 nm in diameter with secondary ~4 nm fibrils protruding—an architecture that couples mesoscale stiffness to nanoscale β-sheets [15]. Diatom β-chitin microfibrils can reach widths up to ~29.8 nm [22], exceeding many fungal α-nanofiber widths and contributing to distinct mechanical/reactivity profiles. Across *Cyclotella* spp., SEM/TEM shows parallel-chain β-paracrystals extruded from fultoportulae pores; Ge-perturbation restricts extrusion but does not alter allomorph [67,69,70,83].

As a rule, fungal α-chitin exhibits smaller crystallites and a higher lattice order than many fungal and microalgal β-systems, whereas diatom β-chitin forms larger-width microfibrils within a parallel-chain framework that trades some crystallinity for intercalation/derivatization capacity [14,15,65,84].

### 3.3. Micro-Morphological Differences

#### 3.3.1. Fungal Chitin

In *Agaricus bisporus*, scanning electron microscopy (SEM) commonly reveals smooth, fibrous surfaces consistent with high-purity α-chitin after alkaline or ionic-liquid routes [29]. Inter-species and tissue-level contrasts (e.g., pileus vs. stipes) are reflected less in direct imaging and more in composition (DA) and crystallinity (CI), which ultimately govern morphological outcomes and enzymatic susceptibility [30]. Mechanical comminution of fresh powders and production residues (Portugal) disrupts the cell wall envelope and improves reagent access; SEM shows similar wall breakage patterns in both streams, indicating convergent microstructures regardless of feed source [42]. SEM on Irish *A. bisporus* wastes shows agglomerated microfibrils rather than the cracked granules typical of chemically over-treated crustacean chitin, pointing to retained fibrillar continuity [38].

Top-down nanostructuring yields characteristic fibrillar morphologies. For chitin nanofibrils (ChNFs) from *A. bisporus*, SEM/TEM/AFM visualizes high-aspect-ratio fibers (tens of nanometers wide; lengths up to ~1 μm), forming entangled networks that remain colloidally stable due to surface chemistry and residual β-glucans [31,33]. Acid hydrolysis of purified mushroom chitin produces chitin nanocrystals (CHNCs) with porous, needle-like particles (TEM ~81 ± 20 nm long, ~19 ± 6 nm wide), reflecting removal of amorphous fractions and cleavage along crystalline lamellae [35]. Compared at equal allomorph (α), fungal ChNCs show larger aspect ratios than shrimp ChNCs and assemble into helicoidal textures at lower solids (1.75 wt% vs. 3.0 wt%), with SEM showing bouligand arches in the visible-color regime [40]. At mesoscales, *A. bisporus* fibers embedded in glucan matrices exhibit water-assisted self-healing after damage, where limited hydration swells and reconnects fibrils, restoring surface smoothness [13]. Classic observations across edible mushrooms (e.g., *Filamentous Ascomycota* and *Lentinula edodes*) report α-chitin nanofibers ~20–28 nm wide with glucan residues that improve aqueous dispersibility [32].

In *Saccharomyces cerevisiae*, engineered activation of the cell wall integrity (CWI) pathway redistributes otherwise septum-biased chitin to a more uniform wall coverage, a micro-architectural shift inferred from staining and consistent with increased load-bearing continuity [12]. In *Pichia*/*Komagataella pastoris*, genetic overexpression thickens and densifies fibril layers without changing α-polymorphy, implying tighter packing and altered porosity rather than a change in crystal type [46]. Yeast-derived CGCs typically appear semi-crystalline and porous after isolation/regeneration; *Penicillium*-line chitosans (while filamentous, often processed similarly) show flaky, irregular platelets by SEM [7]. Adaptive laboratory evolution in *Rhodotorula toruloides* reduces cell size and stiffens walls (flow cytometry/SEM), consistent with higher chitin and mannans contributing to stress-resistant micro-morphologies [55].

In *Fusarium incarnatum*, chitin is integral to the cell wall; yields and wall composition shift with medium (e.g., potato peel vs. wheat bran; yeast extract vs. ammonium sulfate), which is reflected morphologically as differences in wall thickness and fibril density [57]. A soil isolate of *Fusarium incarnatum* places chitin as a major wall fraction (approaching ~40% DW), aligning morphology (dense fibrillar layers) with a structurally dominant role [49]. Chitin–glucan complexes from *A. bisporus* yield porous, smooth films with encapsulation-friendly surfaces [37], while *Pleurotus ostreatus* ChGC exhibits rough, thick surfaces with densely interwoven nanofibers and nanopores suitable for adsorption and tissue scaffolding [81]. Ganoderma lucidum chitin–glucan complex (GLCGC) shows bundled fibrous aggregates; hydrogels form interconnected 3D porous networks whose compactness scales with GLCGC loading [5]. Fungal nanochitin from *Hericium erinaceus* presents ~6.4 nm-wide, ~337 nm-long fibers (aspect ratio > 50:1) [41], while *Mucor indicus* nanofibers are ~28 nm in diameter and uniform after efficient wall disintegration [62]. Cell-level imaging links micro-morphology to synthase genetics: in *A. niger*, chitin–synthase mutants alter septation, branching, and pellet architecture (μCT), with large shifts in pellet diameters and internal hyphal length and wall polymer trade-offs with β-1,3-glucan [58]. Fungal chitosan from *Agaricus bisporus* retains β-glycosidic signatures and hydroxyl bands characteristic of edible mushroom origin [39], and culture optimization can boost *Penicillium* chitosan yields ~3.1-fold, indirectly affecting particle agglomeration and flake size [59].

#### 3.3.2. Microalgal Chitin

Diatoms. *Thalassiosira rotula* secretes extracellular β-chitin microrods via fultoportulae; valve pore position controls rod classes (peripheral pores → ~70 nm diameter, aspect ratio up to ~200; central pores → ~110 nm diameter, aspect ratio ~80), creating two rod populations that bundle and bridge neighboring cells [15]. *Cyclotella cryptica* and *C. meneghiniana* extrude β-chitin nanofibers ~48–58 nm in diameter; high-productivity strains release more fibers per pore (up to ~20.7 in CCMP 333) and longer filaments [50]. In *Cyclotella* sp., β-chitin mats surround frustules; Ge stress distorts biosilica pores and suppresses fiber extrusion, revealing tight coupling between biomineralization and chitin release [67]. Independent lines report β-chitin micro/nanofibers 50–60 nm wide and up to ~100 μm long, secreted as parallel-chain paracrystals [66,68,69,86], and rectangular β-chitin spines up to ~29.8 nm in width [22]. Diatom chitosan from *C. cryptica* retains elongated nanofibrillar morphologies and exhibits lower aggregation than crustacean α-chitosan [65]. Lake systems dominated by centric diatoms (e.g., *Lindavia intermedia*) show thin extracellular β-chitin filaments aggregating into “lake-snow” flocs [72], whereas chlorovirus-infected *Chlorella* forms amorphous extracellular chitin layers rather than ordered nanofibers [73].

Figure 1f-h show marine diatoms from the genera *Thalassiosira* and *Cyclotella*. The images highlight extracellular chitin nanofibers surrounding the cells, demonstrating the structural features of these microorganisms. These pictures are valuable in illustrating how diatoms, with their unique biological machinery, extrude fibers that have different properties compared to those derived from other sources like fungi or crustaceans. This detailed micro-morphology is critical for understanding the potential of diatom-derived chitin in biomedical and material applications, where such structural features can enhance biocompatibility, flexibility, and functionalization.

### 3.4. Causes of Structural Differences

#### 3.4.1. Metabolic Pathway Differences

Mushroom cell walls incorporate α-chitin into β-1,3/1,4/1,6-glucan matrices with outer glycoproteins; this composite architecture biases toward antiparallel packing, higher DA, and semi-crystalline fibrillar morphologies [9,51]. In *Fusarium incarnatum*, chitin synthases localize to hyphal tips to deposit α-chitin microfibrils, differentiating fungal wall biosynthesis from crustacean cuticle assembly [49]. Functional genomics in *Fusarium incarnatum* resolves nine chitin synthases across three clades; domain architecture (e.g., myosin motor domain in CsmA/CsmB) and non-overlapping co-expression networks dictate locus-specific roles (lateral wall deposition, pellet size control, and redundancy revealed in double mutants) that map directly to micro-morphology [58]. In *S. cerevisiae*, deletions in septum/cytoskeletal/trafficking regulators (ACE2, TUS1, CTS1, VTC3, and IRC8) increase wall chitin through pathway-level control [52]. Yeast evolution/engineering further tunes wall architecture: *R. toruloides* upregulates chitin-related genes while down-regulating a chitin deacetylase, reshaping the matrix toward mannans/fucogalactomannans and chitin [55]; *Pichia*/*Komagataella pastoris* boosts UDP-GlcNAc supply (Leloir pathway genes *gfat*, *gna1*, *agm1*, and *uap*) and overexpresses *chs1*/*chs3* to amplify de novo chitin biosynthesis [46].

Diatoms synthesize β-chitin extracellularly at fultoportulae, directly coupling polymerization with secretion; the resulting microrods/fibrils require little to no post-processing, in sharp contrast to fungal wall extraction [15]. Metabolic controls are silica-linked: chitin synthase expression and extrusion co-vary with silicification programs and nutrient status [87]. For green microalgae, stress-responsive activation of biopolymer pathways (e.g., in *Chlorella vulgaris*) enhances wall biosynthesis for adversity adaptation [51].

#### 3.4.2. Environmental Stress Responses

Nitrogen availability modulates chitin accumulation in *F. incarnatum* [49]. In mushroom wastes, alkali pretreatment plus enzymatic hydrolysis increases deacetylation and lowers molecular weight relative to enzyme-only routes, attesting to chemical stress sensitivity [38]. Softer, acid-free extractions in fungi preserve amorphous domains and avoid chain over-degradation compared with crustaceans, a difference rooted in wall biogenesis [10]. High-chitin *S. cerevisiae* mutants pay a growth-rate penalty, revealing a rigidity–proliferation trade-off during stress [52]. In *Zygomycetes*, *nikkomycin Z* selectively perturbs type-c chitin/chitosan synthesis, redistributing hydration and thickening the wall as an adaptive rigidification response [61]. In *A. niger*, stress assays (antifungal proteins, Calcofluor White, and osmotic/thermal challenges) reveal synthase-specific roles in wall integrity and drug responses (e.g., ΔchsE caspofungin resistance and ΔchsF AFP sensitivity) [58]. Evolved *R. toruloides* shows multi-stress tolerance linked to wall remodeling with increased chitin/mannans [55].

In *Cyclotella cryptica*, Si limitation elevates chitin fiber number/length, directly coupling nutrient stress to biopolymer output [65]. Environmental cues modulate “lake-snow” formation in *Lindavia intermedia*, suggesting that P limitation or other stimuli trigger β-chitin fiber secretion [72]. Abiotic Ge stress inserts into biosilica, distorts pore arrays, halts division, and reduces fiber release [67]. Broadly, diatoms upregulate chitin synthases under silica/iron limitation to adjust buoyancy and sinking [87]. In *Chlorella vulgaris*, nutrient deprivation and salinity thicken walls and can triple biopolymer yield while maintaining viability [51].

#### 3.4.3. Comparison with Crustacean Chitin

Fungal feedstocks contain far less mineral (≈2.5–7%) than crustacean shells (≈30–60%), and their chitin is embedded in a chitin–glucan composite rather than a protein–mineral matrix; although yields may be lower, fungal sources are season-independent and sustainable, with fiber strength in the order of 23–28 MPa [20,29]. Microalgal chitin is likewise sustainable and hypoallergenic; in composites, microalgal chitin can enhance barrier and mechanical properties [51]. Diatom β-chitin microrods/fibrils can be harvested intact with largely mechanical/water steps, avoiding the demineralization/deproteinization central to crustacean chitin and preserving native aspect ratios and hierarchy [15]. Fungal isolates such as *F. incarnatum* require only mild alkali/oxidative purification compared with crustaceans, reducing chemical footprint [49]. In mushrooms, stronger alkali reduces yield but increases deacetylation; residues often show slightly higher yields than fresh powders after drying history effects [38]. Meta-analyses emphasize that fungal chitins generally have a lower CI and thermal stability than shrimp α-chitin, yet offer environmental/process advantages and comparable DA-linked functionality [10]. Finally, crustacean chitin is almost exclusively α-type with tight packing and limited intercalation, whereas fungal and microalgal systems, especially diatoms, provide β-type architectures with open galleries suitable for intercalation and derivatization [14].

## 4. Functional Properties of Fungal and Microalgal Chitin

### 4.1. Physicochemical Functional Properties

#### 4.1.1. Solubility and Dispersibility

Enzymatic hydrolysis of cultivation residues reveals species- and tissue-dependent accessibility that correlates with dispersibility in aqueous/enzymatic media: brown *Agaricus bisporus* yielded 31% GlcNAc (*w*/*w*), white *A. bisporus* 28%, while *Pleurotus ostreatus* was much lower at 6.1%, consistent with DA/CI-driven differences in wettability and enzyme penetration [30]. After deacetylation, cap-derived chitosan (Ch-C) from *A. bisporus* displayed higher solubility and more uniform film formation than stalk-derived chitosan (Ch-S). This performance mirrors the higher DA in Ch-C and translates to better dispersion and coating uniformity [43]. Fungal chitin nanofibrils (ChNFs) from *A. bisporus* form stable aqueous colloids owing to nanoscale dimensions and charged/hydrophilic surfaces, which facilitate dispersion without harsh solvents [31,32,33]. Acid-hydrolyzed chitin nanocrystals (CHNCs) from *A. bisporus* disperse well and, when blended into starch matrices, maintain uniform distributions due to extensive hydrogen bonding to polysaccharide hosts [35]. In comparative extractions, *A. bisporus* chitosan showed high solubility in 1% acetic acid (≈72%), exceeding typical *Pleurotus* values, which was attributed to its relatively lower molecular weight [39]. Recent studies on ChNCs-based biocomposite films highlight the importance of ChNC concentration and dispersion methods in modifying the film structure, enhancing mechanical properties, and improving water vapor barrier performance [88].

Native *Komagataella pastoris* CGC is water/solvent-insoluble but becomes highly processable in NaOH/urea systems at subzero temperatures (up to 63–68% solubilization), with urea content being crucial for disrupting H-bonding and, thus, enabling aqueous dispersion routes [53,54]. Recombinant *Pichia pastoris* (GS-3.10) chitin exhibited dispersion behavior comparable to fungal references, with stable suspensions in acidic media suitable for biomedical formulations [46]. Process purity matters: direct deacetylation followed by solution–precipitation, yielded fully soluble chitosan in acetate buffer (pH 4.4), whereas glucan-containing samples showed reduced solubility [89].

Chitosan from *Fusarium incarnatum* (Egyptian soil isolate) dissolves readily in dilute acetic acid, producing stable submicron dispersions amenable to particle engineering [49]. *Pleurotus ostreatus* chitosan is fully soluble in 1% acetic acid (100%), partially in 0.1–0.5% acetic acid (~22–96%), and insoluble in water, H_2_O_2_, and NaOH—profiling a broad acidic dispersibility window [79]. *Aspergillus terreus* chitosan shows good acidic solubility linked to medium Mw (≈54 kDa) and relatively high DD [60]. Optimized Penicillium chrysogenum chitosan (high DD) also shows enhanced acidic solubility against crustacean comparators [59]. Nanochitin from *Hericium erinaceus* disperses stably in water due to surface carboxylates (ζ ≈ −27.8 mV), resisting aggregation [41].

β-Chitosan from *Cyclotella cryptica* exhibits higher solubility in acetic acid than crustacean α-chitosan, reflecting the parallel-chain β-packing that weakens intersheet bonding [65]. Extracellular β-chitin microrods from *Thalassiosira rotula* are expected to disperse readily and assemble into colloids upon gentle agitation [15]. Stress-induced extracellular deposits in *Chlorella* tend to form amorphous layers; once deacetylated, these favor acidic solubilization akin to other fungal and microalgal chitosans [73].

#### 4.1.2. Water Absorption and Retention

*P. ostreatus* chitosan exhibits high water-binding capacity (~527%), attributable to abundant hydroxyl and amino groups [79]. Incorporating *A. bisporus* CHNCs into starch films reduced moisture uptake and water vapor permeability (up to ~1.6–1.9× lower), indicating that α-chitin crystallinity and interfacial H-bonding decrease film hygroscopicity [35]. Water-activated self-healing in *A. bisporus* chitin–glucan fibers demonstrates rapid swelling/deswelling-driven reconnection under microliter water doses [13]. TEMPO-oxidized *Filamentous Ascomycota* (ATC-Sponge) achieved exceptional uptake (~2400% *w*/*w*) via microporosity and carboxyl-mediated hydrophilicity [45]. *A. bisporus* chitosan showed WBC ≈ 674% (vs. 713% for shrimp), underscoring competitive hygroscopicity [39].

Enriched wall chitin in *S. cerevisiae* (CWI activation; high-chitin mutants) suggests enhanced hydration capacity and robust adsorption of chitinases in acidic, ethanol-containing matrices (wine-like systems) [12,52]. Solubilized *K. pastoris* CGC forms viscous solutions with water-retentive networks typical of polysaccharides [53].

*A. terreus* chitosan exhibits WBC ≈ 58.6% suitable for hydrogelation [60]. *Ganoderma lucidum* hydrogels (GLCGCH 1–6) swell massively (≈1181–1891%), with mid-range compositions optimizing porosity vs. structural integrity [5]. Life-cycle assessment suggests that fungal routes can reduce environmental water/energy burdens relative to crustaceans; electricity dominates GWP, inviting gains from renewables/circularity [10].

β-Chitin microrods of *T. rotula* withstand water-based isolation without distortion, consistent with stable hydration shells and retention [15]. In *Mucorales* (β-chitosan-rich walls), water-editing NMR shows chitosan as the most hydrated wall polymer, with chitin/β-glucan relatively dehydrated, an instructive analog for β-rich microalgal assemblies [61].

Nutrient and salinity stresses in *Chlorella vulgaris* thicken walls and can triple biopolymer yields without loss of viability, implying an increased water-binding matrix capacity [51].

#### 4.1.3. Mechanical Properties

**Figure 4:**
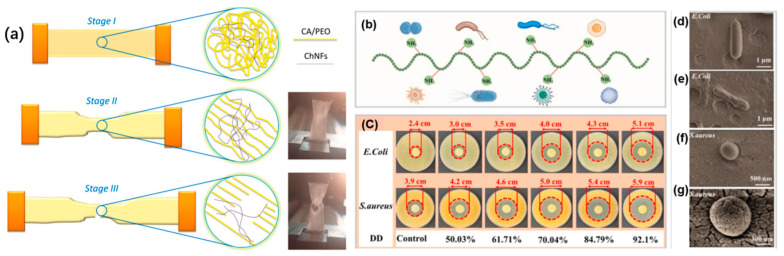
(**a**) Graphical representation of the proposed tensile testing behavior of nanofibrous composites [80]; antimicrobial properties of CBCS: (**b**) schematic diagram of antimicrobial properties of CBCS, (**c**) antimicrobial properties of chitosan with different degrees of deacetylation against *E. coli* as well as *S. aureus*, (**d**) morphology of normally grown *E. coli*, (**e**) morphology of *E. coli* with CBCS added, (**f**) morphology of normally grown *S. aureus*, and (**g**) morphology of *S. aureus* with CBCS added [82].

*A. bisporus* chitin fibers display a tensile strength of 23–28 MPa with 3–5.6% elongation, broadly comparable to shrimp chitin [29]. Chitosan films from mushroom substrates (caps/stalks) showed improved strength/elasticity vs. commercial crustacean analogs, attributable to chain architecture and impurity profiles [43]. Chitin from Irish *A. bisporus* wastes formed stronger films when high-Mw fractions were retained; alkali pretreatment lowered Mw and diminished robustness [38]. Free-standing *A. bisporus* ChNF films reached E ≈ 3.4 GPa, σ ≈ 61.5 MPa ([33]); cellulose-acetate films reinforced with ChNFs showed notable stiffening (E from 200 → 359 MPa) and ~45% higher ductility at 1.5 wt% loading (Figure 4a) [80]. CHNCs (1–7% *w*/*w*) boosted starch-film σ up to 1.7× and E up to 3.9× while reducing strain at break [35]. Water-triggered self-healing yielded tensile/strain recoveries of ~63–119% and ~55–132% within 30 s [13]. Films containing *Suillus luteus* ChNFs achieved σ ≈ 15 MPa with ε ≈ 52% at 5 wt% loading, evidencing simultaneous toughening and flexibilization [85].

Engineered *P. pastoris* increased wall chitin content and packing density, implicating higher local stiffness even with unchanged α-allomorph [46]. Dissolution/regeneration of *K. pastoris* CGC reduced CI and T_d, which typically softens films but improves processability for elastomeric scaffolds [53,54].

*F. incarnatum* chitosan (200 nm–1 μm spheres) offers particulate reinforcement modes in composites [49]. Δ*FvChs3* and related knockouts generated thinner, less stable walls and expanded hyphae—genetic evidence that reduced chitin compromises load-bearing structures [78]. *A. terreus* chitosan exhibited amphiphilic fat-binding (~47.6%), relevant to interfacial mechanics [60]. *Mucor indicus* nanofibers (≈28 nm; high CI) combine nanoscale reinforcement with crystalline stability [62]. Controlled fermentation preserved intact Mw and uniform mechanics in *Mucor rouxii* and *Ganoderma lucidum* chitosans [3,48]. TEMPO-oxidized *P. eryngii* “ATC-Sponge” gained compressive strength via Ca^2+^ cross-linking (~28 → ~175 kPa at 80% strain), tolerating physiological loads in non-compressible wounds [45]. Optical–mechanical coupling in helicoidal f-ChNC films enabled order-of-magnitude reflectance enhancements after alkaline conversion, with tunable visible colors [40].

*Cyclotella cryptica* extrudes longer/more numerous β-chitin nanofibers than *C. meneghiniana*, implying a higher composite reinforcement potential [50]. Single-fibril tests on β-chitin (squid/tubeworm) vs. α-chitin (*Phaeocystis*) report ≈3 GPa vs. ≈1.6 GPa tensile strengths, highlighting the intrinsic advantages of β-packing for load-bearing scaffolds [84]. Rectangular β-microfibrils (widths up to ~29.8 nm) confer rigidity while allowing chemical modification useful for tunable composite interfaces [22]. 

#### 4.1.4. Rheological Properties

Chitin solutions from *A. bisporus* show temperature-dependent viscosity decreases and relative molecular masses comparable to crustacean analogs [29]. Dispersions of nanochitin from *Hericium erinaceus* exhibit shear-thinning with concentration-dependent viscosity, enabling injectable hydrogel design [41]. Curcumin-loaded *A. bisporus* CGC films display disrupted H-bonding, reduced packing/crystallinity, and altered viscoelasticity [37]. Mushroom-derived nanofiber dispersions retain long-term rheological stability, supporting use as thickeners [32]. Solubilized *K. pastoris* CGC solutions show Newtonian plateaus at low shear followed by shear-thinning (η ≈ 11.7–25.2 mPa·s depending on solvent), with G″ > G′ (liquid-like), a favorable profile for extrusion/coating processes [53]. *Ganoderma lucidum* hydrogels (GLCGCH 3–5) exhibit G′ > G″, with gel strength increasing alongside polysaccharide content, consistent with percolated cross-link networks [5]. Although bulk rheology was not measured, the high-aspect-ratio β-microrods/fibers of *T. rotula*/*Cyclotella* suggest field-alignable anisotropic suspensions for electro-/magneto-rheological applications [15,50].

#### 4.1.5. Emulsifying and Colloidal Properties

*A. bisporus* ChNFs’ high aspect ratio and hydrophilic surfaces promote interfacial adsorption and network formation in aqueous colloids; their nanoscale morphology favors Pickering stabilization and film reinforcement [31,80]. Fungal chitin exhibits ampholytic behavior with ζ-potentials from mildly positive (acidic) to negative (alkaline) and an IEP of ~2–4 (near α-chitin ≈ 3.5), much lower than that of chitosan (IEP ≈ 9), enabling pH-tunable colloidal stabilization and water treatment performance [10]. *A. bisporus* chitosan’s fat-binding capacity (~257%) indicates robust interfacial activity for emulsion systems [39]. CGC matrices encapsulate hydrophobes (e.g., curcumin) while densifying microstructure (porosity decreases from ~17% to <1%), thereby enhancing colloidal film stability [37].

High-chitin *S. cerevisiae* cell walls adsorb pathogenesis-related proteins (e.g., chitinases, TLPs) in acidic–ethanolic media, clarifying wine; this demonstrates strong colloidal binding in complex fluids [52]. Solubilized *K. pastoris* CGC produces flowable colloids with shear-thinning behavior suited to biomedical/coating uses [53].

In Mucorales, semi-crystalline chitin interleaved with hydrated chitosan yields dynamic interfaces stabilized by carbohydrate–protein contacts, shaping adsorption and colloidal interactions [61]. *H. erinaceus* nanochitin stability depends on pH/ionic strength (neutral conditions favored; acid/high salt promotes aggregation) [41]. *Mortierella*-derived EPS undergoes sol–gel transitions to stable hydrogels, underscoring colloidal gelation capacity [64].

*Cyclotella* β-nanofibers form dense extracellular colloidal mats; Ge co-addition halves fiber density and weakens network strength, directly linking biomineralization stress to colloidal architecture [66,67,68,69,86]. In culture broths, *Cyclotella* fibers aggregate into adhesive networks; similarly, *Lindavia intermedia* β-chitin filaments assemble into macroscopic “lake snow” flocs that adhere to infrastructure—natural exemplars of strong colloidal cohesion [72]. β-Chitin’s intercalation-friendly galleries further support adsorption/emulsification relative to tightly packed α-chitin [14,65]. Extracellular, amorphous chitin layers around chlorovirus-infected *Chlorella* indicate colloidal coatings distinct from ordered diatom networks, yet still effective for adhesion and encapsulation in aqueous environments [73].

Residual β-glucans commonly present on fungal ChNFs enhance interfacial activity and emulsion stabilization; *Lentinula edodes* β-glucans are effective emulsifiers, suggesting glucan–chitin composites as natural, clean-label stabilizers [32,90]. Moreover, diatom β-fibrils and rectangular microfibrils bring a high surface area and adhesion, complementing fungal α-fibrils in designing colloidal/film systems with tunable charge, wettability, and network mechanics [22,84].

### 4.2. Bioactive Functional Properties

#### 4.2.1. Antimicrobial Activity

Across fungal and microalgal sources, antimicrobial performance depends on polymer size, acetylation pattern, and morphology. In mushrooms, chitooligosaccharides (COSs)—notably chitopentaose and chitohexaose—derived from *Agaricus bisporus* residues inhibit spoilage and pathogenic microbes, supporting food preservation via size- and pattern-dependent interactions with fungal and microalgal membranes and enzymes [30]. Chitosan coatings prepared from *A. bisporus* (cap vs. stalk fractions) suppressed strawberry rots during storage, reducing disease severity from *Botrytis cinerea*, *Penicillium expansum*, and *Fusarium incarnatum* by approximately 60%, 40%, and 30%, respectively; the effect was attributed to chitosan–pectin complexation that impairs pectinases (PG and PME) and cellulases [43]. In composite films, *A. bisporus* ChNFs dispersed homogeneously within CMC/clay matrices, increasing interfacial tortuosity—microstructural features that co-occur with surface activity relevant to antimicrobial barrier functions [91]. Source preparation (freeze-drying vs. supercritical CO_2_) alters powder microstructure but not the basic accessibility that enables subsequent deproteinization/deacetylation, a prerequisite for obtaining active formulations [42]. Mushroom-derived CGC films carrying curcumin were antibacterial against *Escherichia coli* in a dose-dependent manner (no inhibition of *Staphylococcus aureus*), illustrating strain- and formulation-specific outcomes [37]. Fungal α-chitin nanofibers also exhibit inherent antifungal and microalgal utility in wound contexts through barrier and moisture management effects [32].

Yeast- and fungus-derived chitosans show broad-spectrum action. Submicron chitosan from *Fusarium incarnatum* (Egyptian soil isolate) inhibited *E. coli* and *S. aureus*, with particle-scale morphology aiding contact killing and film formation [49]. Chitosans isolated from *Penicillium* sp. IITISM-ANK1 and *P. johnkrugii* IITISM-ANK2 produced inhibition zones against *E. coli* in disk assays [7]. Figure 4b illustrates the antimicrobial activity of CBCS (from *Flammulina velutipes* waste) at different degrees of deacetylation (DDs) against *E. coli* and *S. aureus*. As the DD increases, the inhibition zone diameter enlarges, indicating enhanced antimicrobial efficacy (panel b). SEM images (panels c–f) show significant morphological changes in both *E. coli* and *S. aureus* after CBCS treatment, suggesting that a higher DD enhances bacterial membrane disruption and overall antibacterial activity [82]. In *Filamentous Ascomycota*, unbleached chitosan (U.Cs.Pe) achieved MICs of 0.375 mg/mL (*Clavibacter michiganensis*) and 0.75 mg/mL (*Monilinia laxa*) and outperformed a commercial reference [36]. Engineered *Pichia pastoris* (GS–3.10) generated chitin with preliminary antimicrobial behavior comparable to fungal chitosan [46].

**Figure 5:**
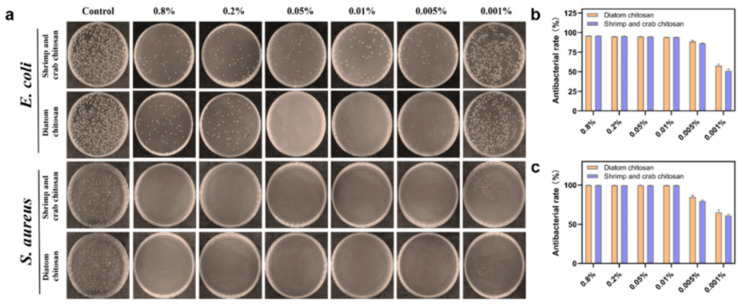
(**a**) In vitro antibacterial activity images of diatom chitosan and shrimp and crab chitosan. (**b**) Statistical results of *E. coli*. (**c**) Statistical results of *S. aureus* [65].

Among microalgae, β-type chitosan from *Cyclotella cryptica* (diatom) achieved >90% inhibition of *E. coli* and *S. aureus* at 0.005%, paralleling crustacean benchmarks (Figure 5) [65]. For diatom β-chitin nanofibers/microrods (e.g., *Thalassiosira rotula*), a high aspect ratio and open-sheet packing support aqueous dispersion and interfacial adhesion that can potentiate antimicrobial composite design [15].

#### 4.2.2. Biocompatibility

Fungal and yeast chitins are generally biocompatible and non-allergenic alternatives to marine sources. Yeast α-chitin from *Saccharomyces cerevisiae* is structurally indistinguishable from crustacean α-chitin and, thus, is expected to be cytocompatible [12]. *Fusarium incarnatum* chitin—free of shellfish allergens—meets food/biomedical compatibility expectations [92]. Submicron *F. incarnatum* chitosan showed no mammalian cytotoxicity, supporting medical use [49]. Films reinforced with *Suillus luteus* ChNFs were non-cytotoxic, suitable for wound or food-contact packaging [85]. In CMC/clay films, *A. bisporus* ChNFs reduced transparency but markedly improved UV shielding, attributes relevant to protecting photosensitive payloads while maintaining safety [91]. Extraction variables (NaOH concentration/time) modulate DA without introducing cytotoxic solvents; in *A. bisporus* powders and residues, higher alkali lowered yield but raised DA, with minimal differences in DA arising from supercritical CO_2_ pretreatment [42]. Low-ash, α-type *A. bisporus* waste chitin also demonstrated biomedical compatibility [38]. Diatom *C. cryptica* chitosan sustained >95% L929 fibroblast viability at 20 mg/mL, exceeding shrimp/crab analogs [65]. *A. bisporus* ChNFs and f-ChNCs were cytocompatible with human/mouse cell lines up to 5 mg·mL^−1^ and pose lower allergenic risk than crustaceans [31,33]; water-triggered self-healing of *A. bisporus* fibers further underscores a solvent-minimal, biocompatible pathway [13]. Oxidized ATC-Sponge from *Filamentous Ascomycota* maintained >100% cell viability and <5% hemolysis, with in vivo implantation showing resolution of acute inflammation [45]. *Ganoderma lucidum* hydrogels preserved ~100% fibroblast viability with hemolysis 0.9–1.5% [5]. Fungal COs from *P. ostreatus*, *Cunninghamella bertholletiae*, and *Trichoderma viride* acted as symbiotic signals (robust nuclear Ca^2+^ oscillations in *Medicago truncatula*), outperforming shrimp COs [74]. Purified *P. ostreatus* ChGC was biodegradable/biocompatible with low protein carryover [81]. Ecologically, *Lindavia intermedia* lake-snow fibers restructure trophic dynamics without apparent toxicity [72], while *Cyclotella* β-chitin is a tunable, renewable biomedical feedstock [67]. Fungal chitosans produced with controlled fermentation (e.g., *Mucor rouxii* and *G. lucidum*) afford batch consistency and high DDA aligned with medical requirements [3,34,48,89]. *Chlorovirus*-induced *Chlorella* chitin further illustrates benign phototrophic biopolymer generation [73]. β-Chitin’s low protein/mineral burden reduces immunogenic risk relative to shellfish sources [14].

#### 4.2.3. Biodegradability

Fungal and microalgal chitins retain enzymatic degradability through chitinases/lysozymes, enabling environmental compatibility and resorption in vivo. Yeast *S. cerevisiae* α-chitin preserved natural biodegradability [12], and *A. niger* α-chitin remained enzyme-susceptible [92]. *F. incarnatum* chitosan degraded progressively under enzymatic conditions [49]. HPMC–FA films with *Suillus* ChNFs were environmentally degradable [85]; *A. bisporus* ChNFs likewise fully biodegrade and are applicable to green packaging and biomedicine [31]. ATC-Sponge (chitin/glucans) is fully resorbable in vivo, avoiding removal-related trauma associated with non-degradable hemostats [45]. Waste-derived *A. bisporus* chitins preserved biodegradability comparable to crustaceans and allowed controlled degradation for tissue engineering [38]. Developmental cycles in fungi natively modulate CGC turnover, underscoring intrinsic biodegradability [56]. Processing nuances that change DA/CI (e.g., in *A. bisporus* powders/residues) do not eliminate biodegradability, though they tune rates [42].

#### 4.2.4. Antioxidant Activity

Mushroom-derived COSs act as radical scavengers with activity governed by DP and acetylation motifs [30]. In fresh produce, *A. bisporus* chitosan coatings curtailed weight loss (15.6% → 11.1%) and preserved firmness, TAA, TPC, and AsA, consistent with barrier-mediated oxidative protection [43]. In CMC/clay matrices, *A. bisporus* ChNFs maximized tensile performance (e.g., CMCC-NC20 σ ≈ 29.2 MPa; E ≈ 1120 MPa) while contributing antioxidant reinforcement through hydrogen-bonded, electrostatically interactive networks [91]. Extraction histories that induce α→β polymorph shifts (supercritical CO_2_) or change CI/DD (alkali strength) alter electron-donating capacity indirectly via crystallinity and accessible functional groups [42]. Curcumin-loaded *A. bisporus* CGC films achieved up to ~71% radical-scavenging activity, while even unloaded CGC films retained baseline activity from intrinsic mushroom phenolics [37]. Among filamentous fungi, *Mortierella alpina* EPS showed strong ABTS scavenging (IC_50_ ≈ 2.08 mg·mL^−1^; TEAC ≈ 989 μmol·g^−1^), surpassing many crustacean/insect references [64]. *Ganoderma lucidum* chitosan displayed antioxidant capacity on par with marine analogs [3]. Mushroom β-glucans (e.g., *Lentinula edodes*) furnish additional antioxidant activity and synergize with chitinous scaffolds in composites [90]. Diatom-derived β-chitin/chitosan maintains high purity (low ash/protein), supporting clean redox profiles and minimizing pro-oxidant impurities [14], while cultivation controls (e.g., Ge stress and Si limitation) tune fiber production without introducing cytotoxic byproducts [65,67].

#### 4.2.5. Antitumor and Anti-Inflammatory Activities

Mushroom-derived chitooligosaccharides (COSs) display size- and pattern-dependent bioactivity; specific acetylation motifs are linked to both anti-inflammatory signaling and anticancer potential [30]. *Pleurotus ostreatus* chitosan inhibited proliferation of MDA-MB-231 breast cancer cells with an IC_50_ ≈ 10 μg·mL^−1^; flow cytometry and RT-PCR evidenced apoptosis (early/late) with caspase-3 up-regulation [79]. Free-standing chitin nanofibrils (ChNFs) from *Agaricus bisporus* were non-cytotoxic in standard viability assays, yet elicited dose-dependent nitrite and TNF-α release in BV-2 microglia (1–5 mg·mL^−1^), underscoring immunomodulatory potential and the need for careful dose/formulation design [33]. In CMC/clay films, A. bisporus ChNFs adjusted the surface wettability, with the contact angle initially decreasing at low loading concentrations, followed by an increase to approximately 46° at a 30 wt% loading. This change in wettability is important for macrophage adhesion and phenotype modulation at biomaterial interfaces [91]. Mushroom β-glucans further contribute immunomodulatory/antitumor activity by activating macrophages/lymphocytes [90].

Engineered *Pichia pastoris* (GS–3.10) produced chitin with preliminary antimicrobial/biomedical parity to fungal chitosan, suggesting potential anticancer/anti-inflammatory utility once processing to COS/chitosan is optimized [46]. *Mortierella alpina* extracellular polysaccharide (EPS) selectively inhibited multiple tumor lines (H295R, CACO-2, MDA-MB-231, MDA-MB-468, and MCF7) by >50% at 1.3–2.1 mg·mL^−1^ while sparing VERO/MCF10A cells [64]. Extracts of *Ganoderma lucidum* chitosan also showed selective cytotoxicity in cancer-related models, aligning with its long-noted biomedical relevance [3].

#### 4.2.6. Phytotoxicity and Cytotoxicity

In soil burial, *A. bisporus* ChNFs accelerated film biodegradation (CMCC-NC30 ≈ 52.1% mass loss in 4 weeks vs. 37.3% for clay-only CMCC), indicating enhanced fungal and microalgal accessibility without added phytotoxicity [91]. Fungal ChNFs were less cytotoxic than cellulose nanomaterials but can trigger microglial inflammatory signaling, highlighting application-dependent safety thresholds [33]. COSs from *P. ostreatus* promoted arbuscular mycorrhizal colonization in *Medicago truncatula* with no phytotoxic signs, outperforming shrimp-derived counterparts [74].

#### 4.2.7. Bioactive Encapsulation and Oxidative Stability

Incorporation of *A. bisporus* ChNFs endowed otherwise inactive CMC/clay films with antibacterial function (inhibition zones of 1.56–2.62 mm at 30 wt%, strongest vs. *E. coli*), while simultaneously reinforcing mechanics via hydrogen bonding/electrostatics—features favorable for active packaging or wound dressings where oxidative stability and barrier properties matter [91].

β-Chitin succinate from *Thalassiosira weissflogii* templated periodic Pb^2+^ arrays, evidencing ordered coordination sites that can be translated to encapsulate bioactives/ions with spatial regularity [17].

#### 4.2.8. Hemostatic Activity

Oxidized, Ca^2+^-complexed *Filamentous Ascomycota* ATC-Sponge showed rapid blood-triggered shape memory (5–8 s), with dramatic hemostasis in rat femoral artery and liver puncture models (blood loss ~198 ± 16 mg and ~82 ± 22 mg, respectively), attributable to high water uptake, platelet/RBC aggregation, fibrin formation, and Ca^2+^-accelerated coagulation [45]. *Ganoderma lucidum* hydrogels (GLCGCH 3–5) achieved clotting indices of 35.3–41.9%, comparable to a gelatin sponge, far superior to untreated GLCGC (71.6%); porosity and chitin–glucan synergy drove rapid cell adhesion and clot nucleation [5]. Curcumin-loaded *A. bisporus* CGC films coupled antioxidant/antibacterial functions relevant to wound settings, consistent with the broader role of fungal chitin in hemostatic dressings [37]. Chitin nanofibers from edible mushrooms promote fast coagulation and wound closure, supporting advanced hemostat development [32]. Sustainability co-benefits are non-trivial: adding *A. bisporus* ChNFs to packaging films modestly lowered life cycle impacts relative to clay-only composites while delivering antibacterial function [91].

*Cyclotella cryptica* chitosan shortened clotting time in vitro (≈430 s vs. ≈480 s for shrimp/crab, ≈600 s control) and markedly reduced blood loss in vivo (rat tail transection), matching marine references [65]. The combination of β-chitin’s open structure and high purity offers rapid hydration and platelet interaction pathways comparable to state-of-the-art animal sources [14,65].

### 4.3. Core Mechanisms of Structure–Function Relationships

#### 4.3.1. Molecular-Level Mechanisms

In *Agaricus bisporus*, high-purity extractions yield smooth fiber surfaces and higher crystallinity, which translate into enhanced thermal stability and predictable solvent response [29]. The bioactivity of mushroom-derived COSs is governed by an interplay among DP, DA, and PA; these molecular descriptors dictate recognition by hydrolytic enzymes and host receptors and, therefore, modulate antifungi and microalgae, anti-inflammatory, and antitumor outcomes [30]. Water-activated “self-healing” in *A. bisporus* fibers arises from reversible hydrogen bonding between α-chitin fibrils and β-glucans; transient swelling aligns fibrils and restores load-bearing capacity, consistent with full or supra-baseline recovery of strength/strain in thicker fibers [13]. A hybrid chitin/β-glucan nanofibril architecture with abundant hydrogen-bond donors/acceptors explains both the mechanical robustness of *A. bisporus* chitin nanofibrils (ChNFs) and their measurable innate immune reactivity [33]. More broadly across saprotrophic taxa, ecological adaptation and carbon use appear to bias wall biogenesis towards higher chitin accumulation (e.g., *Ganoderma lucidum* and *Lentinula edodes*) compared with several cultivated or wood-destroying strains, indicating lineage-specific regulation of precursor flux into chitin synthesis [8].

In *Saccharomyces cerevisiae*, constitutive activation of the RHO1–PKC1 cell wall integrity (CWI) pathway re-routes carbon–nitrogen flux towards chitin biosynthesis, elevating wall chitin without exogenous stressors; at the molecular level, this rewiring increases chitin deposition beyond the bud neck/septum to a more uniform wall distribution [12]. Engineered *Pichia*/*Komagataella* platforms similarly boost UDP-GlcNAc supply and chitin synthase activity, providing tunable chitin chain length and density that carry forward into solution behavior and downstream derivatization [46].

In *Fusarium incarnatum*, nutrient regime (C/N ratio and protein supplementation) and cultivation time couple directly to chitin yield and wall composition; optimized conditions (e.g., wheat-bran/yeast-extract media, 10 d) maximize biomass and chitin fraction, linking macromolecular output to primary metabolism [57]. Genetic dissection supports a compensation model among chitin synthases: loss of one synthase (e.g., *csmA*) is offset by over-contribution of others under shear, shifting the chitin–glucan balance; reduced septal chitin across knockouts implies a non-redundant, multi-enzyme requirement at septa, whereas *chsE*/*chsD* exhibit conditional redundancy along the lateral wall [58]. In *Fusarium incarnatum* (Egyptian soil isolate), chemical deacetylation of chitin to chitosan followed by ionotropic gelation (e.g., tripolyphosphate) yields submicron particles; DA reduction increases amine density, enabling electrostatic cross-linking and the emergent particle mechanics and bioactivity associated with nanoscale chitosan [49]. Stress-adapted remodeling in *Rhodotorula toruloides* increases β-linked polymers (glucomannans and fucogalactomannans) and chitin while down-tuning (1→4)-glucans; concurrent gains in GPI-anchored sensors amplify CWI signaling and wall rigidity [55].

In *Thalassiosira rotula*, β-chitin chains assemble into ~16–20 nm nanofibrils that pack hierarchically into microrods; occasional ~4 nm secondary fibrils protrude from rod surfaces, revealing a multiscale build-up from molecular chains to mesoscale rods that underpins a high aspect ratio and colloidal assembly [15]. Relative to α-chitin, diatom β-chitin features weaker intersheet hydrogen bonding and open galleries that facilitate intercalation and derivatization without extensive depolymerization [14].

Although less structurally resolved than diatoms, stress-programmed wall biogenesis in *Chlorella* increases chitinous and complementary polysaccharide layers, supporting adversity tolerance and defining the chemical landscape for subsequent deacetylation/derivatization [51].

#### 4.3.2. Microscopic-Level Mechanisms

In *Agaricus bisporus*, crystallite size and orientation (e.g., α-chitin (020)/(110) domains) correlate with stiffness and thermal stability, providing a direct microstructure-to-property link for fibers and films [29]. At the micron scale, water ingress activates reversible H-bond re-formation between chitin fibrils and β-glucans, enabling rapid self-healing that restores continuous load paths across fractured interfaces [13]. The coexistence of chitin with covalently/physically bound β-glucans produces hybrid nanofibrils of a high aspect ratio that form entangled meshes; these meshes govern permeability, protein adsorption, and innate immune cell engagement at biomaterial interfaces [33].

Uniform wall deposition achieved via CWI activation in *S. cerevisiae* increases the density of α-chitin microdomains across the cell surface, altering porosity and adsorption sites and thereby shifting colloidal and filtration behavior of the biomass [12]. In engineered *Pichia*/*Komagataella*, thicker and denser fibril layers reported under stress culture suggest tighter packing and altered defect density within α-microfibrils—features that influence stiffness, swelling kinetics, and dissolution routes [46].

Three-dimensional µCT of *A. niger* pellets links specific synthases to pellet core architecture—hyphal length density, branching frequency, growth, and branch units—demonstrating that chitin synthesis programs set diffusion-relevant microstructures that regulate oxygen/nutrient penetration and, downstream, productivity in submerged culture [58]. Media-driven shifts in wall composition and stress-responsive remodeling further tune hyphal thickness [55,92], pore tortuosity, and mechanical anisotropy—microstructural variables that propagate up to filtration, rheology, and composite reinforcement performance. In *F. incarnatum*, nanoscale chitosan formation via ionotropic gelation introduces particulate architectures with high surface-to-volume ratios, changing interfacial mechanics and enabling contact-killing/adsorptive functions in films and coatings [49].

The number and spatial distribution of fultoportulae pores on silica valves program the geometry of secreted β-chitin: *T. rotula* typically deploys ~100 peripheral and ~16 central pores per valve, yielding two distinct microrod populations (narrow/high-aspect peripheral vs. wider/lower-aspect central rods). This hard-coded pore map governs rod diameter, length, and bundling, thereby controlling colloidal networking, floc formation, and interparticle adhesion during aqueous processing [15]. The β-allomorph’s open galleries further permit post-secretion intercalation and ion coordination at the rod surface without collapsing the crystalline core, preserving mechanical integrity while adding chemical functionality [14].

In *Chlorella vulgaris*, alternating wall strata—electrostatically complementary (negatively charged cellulose-rich layers and positively charged chitinous layers)—increase barrier strength and tune hydration/ion transport; under nutrient or salinity stress, these lamellae thicken, elevating biopolymer yield and redefining interfacial charge for downstream processing [51]. Collectively, these micro-architectures explain how organism-specific biosynthetic programs (fungal septal vs. lateral wall, yeast CWI redistribution, diatom pore-templated extrusion, and green-algal lamellar stacking) translate molecular variables (DP, DA, PA, and allomorph) into a mesoscale structure and, ultimately, into physicochemical and bioactive functions [8,12,13,14,15,29,30,33,49,51,55,57,58].

## 5. Biomedical Applications of Fungal and Microalgal Chitin

Similarly to other polysaccharides, fungal and microalgal chitin has demonstrated a wide range of applications in the field of biomedicine [1,2,93,94,95]. Fungal and microalgal-derived chitin offers a broad spectrum of biomedical translation prospects, supported by its structural tunability, biocompatibility, and sustainable production routes. Evidence from mushrooms, yeasts, filamentous fungi, and diatoms points to several concrete application domains:

### 5.1. Wound Healing and Hemostatic Dressings

The use of chitosan in biomedical applications is vast, particularly in drug delivery systems. Chitosan’s unique properties, including its ability to modify surface characteristics, make it an excellent candidate for controlled drug release. By adjusting its molecular structure, chitosan can be tailored to release therapeutic agents in a specific, targeted manner, allowing for precise control over the rate and location of drug delivery [96]. This functionality is crucial for enhancing the efficacy of treatments while minimizing side effects, making chitosan a key material in advancing pharmaceutical delivery systems.

**Figure 6:**
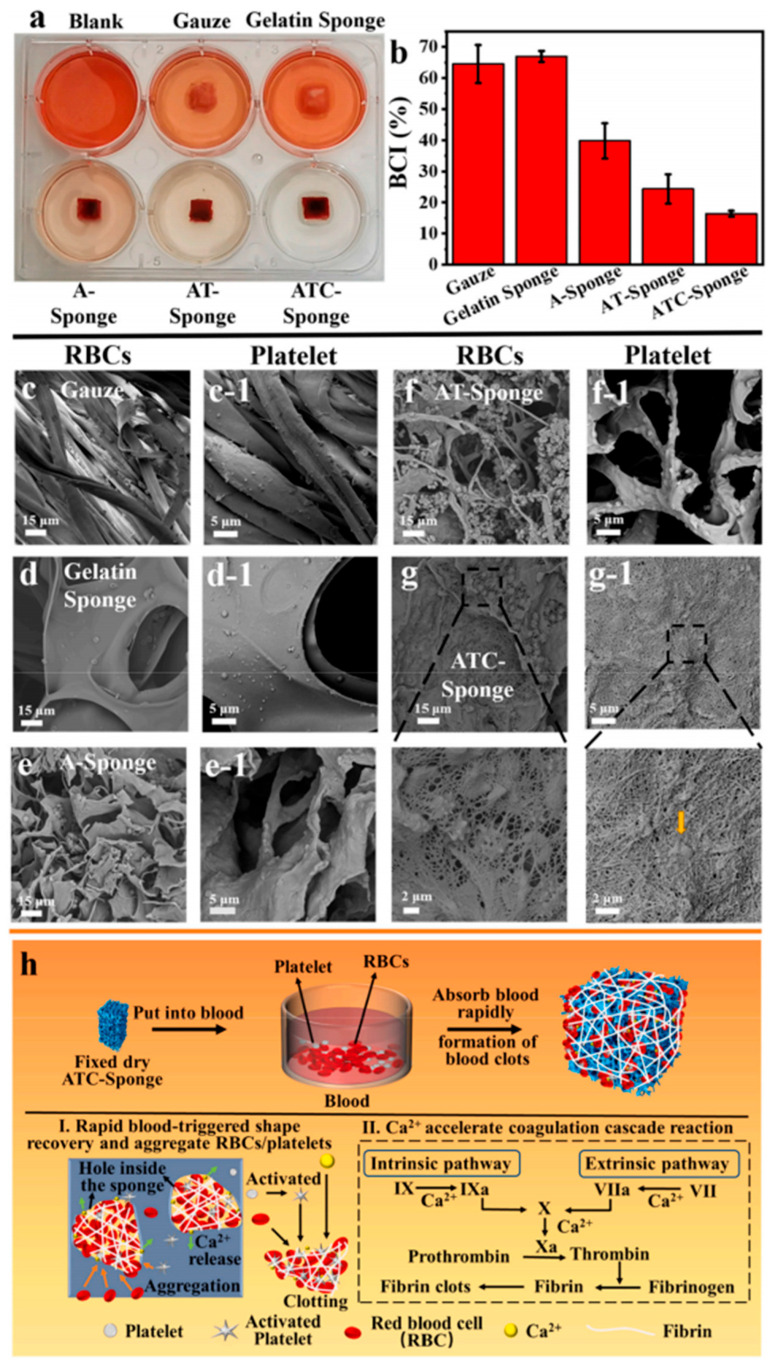
In vitro coagulation performance assessment: (**a**) digital photos of whole-blood-clotting and (**b**) BCI of gauze, gelatin sponge, A-Sponge, AT-Sponge, and ATCSponge. SEM images of the coagulation behavior on the hemostasis materials: (**c**,**c-1**) gauze; (**d**,**d-1**) gelatin sponge; (**e**,**e-1**) A-Sponge; (**f**,**f-1**) AT-Sponge; and (**g**,**g-1**) ATC-Sponge. (**h**) Hemostatic mechanism of ATC-Sponge [45].

In addition to drug delivery, chitosan-based materials derived from fungi and diatoms have shown great promise in the field of wound healing. These materials demonstrate robust biological activity, particularly in promoting tissue regeneration and accelerating the healing process. For example, chitin nanofibrils and hydrogels derived from mushrooms provide highly breathable wound dressings that are not only mechanically strong, but also have the ability to self-heal. This self-healing feature is activated when the material is exposed to moisture, which helps to repair any structural damage, extending the dressing’s lifespan without compromising its functionality (Figure 6) [13,45]. Additionally, composites like the *ATC-Sponge*, which combines chitin and glucan components, show rapid shape-memory expansion. This property allows the sponge to quickly conform to wound shapes, significantly reducing blood loss during in vivo applications, making it highly effective for managing trauma [45].

Fungal-based materials, such as hydrogels derived from *Ganoderma lucidum*, have demonstrated clinically relevant hemostatic properties. These hydrogels promote clot formation and accelerate the coagulation process, making them highly effective in stopping bleeding in traumatic injuries. The excellent compatibility of these materials with blood cells (hemocompatibility) ensures that they do not induce adverse immune responses when used in wound care [5]. Similarly, chitosan extracted from *Cyclotella cryptica*, a species of diatom, has also been shown to significantly enhance blood clotting and promote faster hemostasis. These findings highlight the importance of fungal and microalgal chitin as advanced materials in trauma care, particularly for surgical applications where rapid bleeding control is critical [65].

Collectively, these studies underscore the potential of microbial fungal and microalgal chitin-based materials as innovative solutions for improving trauma care and surgical outcomes. Their unique combination of hemostatic properties, biocompatibility, and mechanical strength positions them as key materials for developing advanced wound healing products and surgical treatments. These findings are supported by further research that highlights the growing importance of these materials in the biomedical field [97,98] (Table 3).

### 5.2. Drug Delivery and Controlled Release Systems

Fungal and microalgal chitosan and nanofibrils act as carriers for controlled drug release. Submicron chitosan from *Fusarium incarnatum* provides antimicrobial and cytocompatible nanoparticles for wound dressings and DDSs [49]. Regenerated yeast chitin–glucan complexes enable aqueous processing into fibers and films suited for therapeutic encapsulation [53]. Diatom β-chitin, particularly in succinate-modified form, demonstrates ion templating capacity for ordered molecular encapsulation, offering routes for precise drug delivery [15,17].

Fungal and microalgal chitosan, along with chitin nanofibrils, are being explored as carriers for controlled drug delivery systems (DDSs) due to their excellent biocompatibility, biodegradability, and ability to modify surface properties, which are crucial for precise drug release [49]. The use of submicron chitosan, particularly derived from *Fusarium incarnatum*, has been shown to improve antimicrobial activity and enhance cytocompatibility, making it an ideal candidate for wound dressings and DDS applications. The ability of these nanoparticles to interact effectively with wound surfaces facilitates the targeted delivery of drugs, ensuring that therapeutic agents are released at the right site and in the correct amounts, thus minimizing side effects [49].

In addition, the unique properties of yeast-derived chitin–glucan complexes allow for the creation of fibers and films that can be processed in aqueous environments, opening the door to their use in therapeutic encapsulation applications. These materials demonstrate enhanced solubility compared to chitin alone, which is often hindered by its inherent insolubility in most solvents [53]. The biodegradable nature of these complexes also means they can be safely used in medical applications without posing long-term environmental concerns, making them suitable for various pharmaceutical uses.

Another promising advancement in drug delivery systems involves the use of diatom-derived β-chitin, particularly in its modified succinate form. This form of β-chitin is unique in that it possesses ion templating properties, which allow for the encapsulation of molecules in a highly ordered manner. The succinate modification introduces carboxyl groups that facilitate the ion exchange of metal cations, which can be useful in drug delivery for applications that require precise molecular encapsulation. The ion templating properties of this material offer new pathways for controlled drug release, as the encapsulated molecules can be released in a controlled fashion in response to environmental stimuli [15,17]. The ability to fine-tune these properties opens up avenues for designing systems that are responsive to specific physiological conditions, such as changes in pH or ionic strength, which can trigger the release of drugs in targeted areas of the body.

Additionally, the development of chitin-based nanofibrils from marine diatoms and fungi, with their high aspect ratios and structural integrity, has also shown promising results in drug encapsulation. These materials can be easily functionalized and modified for various biomedical applications, including sustained and controlled drug release, which is crucial for treatments requiring long-term administration [15]. This aspect of chitin-based nanomaterials makes them a significant addition to the growing field of biomaterials designed for advanced drug delivery.

In summary, the versatility of fungal and microalgal chitin-based materials, including their ability to encapsulate and release drugs in a controlled manner, positions them as innovative solutions in the field of drug delivery. These materials not only offer a sustainable alternative to synthetic polymers, but also provide superior performance due to their unique chemical and physical properties [15,17,53].

### 5.3. Tissue Engineering and Biomedical Scaffolds

Chitin nanofibrils and nanocrystals from mushrooms (*Agaricus bisporus*, *Mucor indicus,* and *Hericium erinaceus*) form strong, porous scaffolds with tunable mechanical and hydrophilic properties, ideal for tissue regeneration [33,41,62]. Diatom β-chitin nanofibers and microrods offer high aspect ratios and a hierarchical architecture, further supporting scaffold design [14,67]. Their biodegradability ensures compatibility for temporary implants and regenerative systems.

ChNFs and nanocrystals, derived from various fungal species such as *Agaricus bisporus*, *Mucor indicus*, and *Hericium erinaceus*, have shown remarkable potential as materials for tissue regeneration due to their ability to form strong, porous scaffolds with tunable mechanical and hydrophilic properties [33]. These properties make them ideal for applications where the structure and performance of the scaffold play a critical role in supporting tissue growth and healing. Chitin-based scaffolds are inherently biocompatible and support cellular attachment, proliferation, and differentiation, which are key aspects for tissue engineering and regenerative medicine [62].

The structural attributes of chitin nanofibrils, such as their high surface area and nanoscale dimensions, allow for the design of scaffolds with tailored mechanical strength and surface chemistry. These attributes can be optimized to meet the specific demands of different types of tissue regeneration, such as skin, cartilage, and bone. Moreover, the hydrophilic nature of these nanomaterials enhances their ability to retain water, mimicking the natural extracellular matrix (ECM) that supports cell growth and function. Studies have shown that chitin scaffolds made from *Agaricus bisporus* and other fungi not only provide mechanical support, but also encourage the formation of new tissue by acting as a template for cell migration and attachment [33]

Additionally, diatom-derived β-chitin nanofibers and microrods offer unique advantages due to their high aspect ratios and hierarchical architecture. These characteristics allow for enhanced mechanical stability and the creation of highly structured, scalable scaffolds [67]. Diatom-based chitin materials are known for their well-organized nanostructures, which can be crucial in replicating the complexity of biological tissues. These nanofibers also provide an increased surface area for cellular interactions, which can further support the attachment and growth of cells within the scaffold, enhancing tissue regeneration.

The biodegradability of these chitin-based materials is another crucial feature that makes them particularly suitable for regenerative medicine. Their natural decomposition over time ensures that they do not leave harmful residues within the body, reducing the risk of long-term complications associated with synthetic materials. As these scaffolds break down, they can be replaced by the regenerating tissue, promoting natural healing processes. This characteristic makes chitin-based scaffolds an excellent choice for temporary implants in regenerative systems, especially in applications where the material must be resorbed as the tissue heals [41].

The inherent properties of fungal and diatom chitin, including their mechanical strength, biodegradability, and hydrophilic nature, position them as advanced materials for applications in both tissue engineering and temporary implants. The ability to modify the physical and chemical characteristics of these materials opens up new possibilities for designing scaffolds tailored to specific tissue types, enhancing the success of regenerative treatments and improving clinical outcomes [14,33]. These findings underscore the growing importance of chitin nanofibrils and nanocrystals in the development of sustainable and effective biomedical applications, particularly in the field of tissue regeneration and repair.

### 5.4. Antimicrobial and Antioxidant Biomedical Coatings

Mushroom-derived chitosan films, especially from *Agaricus bisporus*, exhibit strong antimicrobial activity against common food-borne and wound pathogens, such as *Escherichia coli* and *Staphylococcus aureus* [37]. These films provide an eco-friendly solution for food packaging, where their ability to prevent microbial growth extends the shelf life of food products. Additionally, the incorporation of bioactive compounds like curcumin enhances the films’ antioxidant properties, offering protective effects against oxidative damage [37]).

Films reinforced with *Suillus luteus* chitin nanofibers further enhance these properties, displaying antibacterial, antioxidant, and controlled-release characteristics. These films are suitable for both biomedical coatings and active packaging, offering dual clinical and industrial applications [85,99]. Their biodegradability and strength make them ideal for temporary implants and sustainable packaging, benefiting from both mechanical reinforcement and bioactive agent release.

### 5.5. Anticancer and Anti-Inflammatory Therapies

Fungal and microalgal chitin derivatives have shown selective anticancer activities. For example, chitosan from *Pleurotus ostreatus* suppresses MDA-MB-231 breast cancer cell proliferation by inducing apoptosis and activating caspase-3 [79]. This suggests its potential as a selective cancer therapy, reducing the toxicity associated with traditional treatments.

Exopolysaccharides from *Mortierella alpina* also exhibit selective inhibition of tumor cells while sparing normal cells, highlighting their potential as an adjuvant cancer therapy [64]. Furthermore, mushroom-derived β-glucans, such as those found in *Lentinula edodes* (shiitake mushrooms), enhance immune responses and show anti-inflammatory effects, further supporting their role in anticancer therapies [90].

These findings position fungal and microalgal chitin derivatives as promising, eco-friendly alternatives in cancer treatment, offering both direct anticancer effects and immune system modulation.

### 5.6. Biodegradable and Sustainable Medical Materials

Fungal and microalgal chitin derivatives are gaining attention for developing biodegradable packaging and eco-friendly biomaterials, which help reduce clinical and environmental waste. Mushroom-derived nanofibrils, for example, improve the barrier and mechanical properties of films while maintaining biodegradability in soil, making them ideal for sustainable packaging [37,91]. These films offer an effective alternative to traditional plastics, breaking down naturally without harming the environment.

Diatom-derived β-chitin, obtained with minimal chemical treatments, is another promising material for eco-friendly scaffolds and composites [15]. This environmentally benign approach ensures that the materials can be used for both biomedical and industrial applications without contributing significantly to waste. The ability of diatom chitin to be extracted sustainably from algae further enhances its potential as a resource for eco-friendly products [17].

These advances make fungal and microalgal chitin essential components in developing materials that support both environmental sustainability and the reduction in waste.

## 6. Conclusions

Fungal and microalgal chitin has emerged as a promising alternative to traditional crustacean sources, offering advantages of renewability, reduced allergenicity, and scalable production. Recent studies have highlighted its unique structural polymorphism, high biocompatibility, and diverse bioactivities, supporting applications ranging from biomedicine and food preservation to sustainable packaging and environmental remediation. Compared with crustacean chitin, fungal and algal sources require simpler demineralization, generate lower environmental burdens, and can be cultivated on agro-industrial residues, aligning well with the principles of the circular bioeconomy.

Despite these advances, challenges remain in achieving cost-effective production, standardized safety evaluation, and clinical validation. Future progress will rely on integrating green extraction technologies, strain engineering, and interdisciplinary applications to unlock the full translational potential of fungal and microalgal chitin. With continued innovation, fungal and microalgal chitin is poised to evolve from a niche biopolymer into a mainstream sustainable material with broad impacts across medicine, agriculture, and industry.

## 7. Limitations and Future Perspectives

Although fungal and microalgal chitin shows remarkable potential as a sustainable and versatile biomaterial, several limitations remain. Current studies are largely confined to laboratory-scale evaluations, with insufficient evidence from clinical trials to confirm safety and efficacy. Standardized protocols addressing toxicology, immunogenicity, and biodegradation are still lacking. Moreover, production remains challenged by low yields, high enzyme demand, and costly downstream processing, limiting industrial feasibility.

Future work should focus on establishing clear structure–function correlations, developing green and scalable extraction strategies, and expanding the diversity of fungal and microalgal sources through synthetic biology and metabolic engineering. In parallel, efforts to validate biomedical applications through in vivo models and clinical studies are essential. Addressing these gaps will be critical for translating fungal and microalgal chitin from promising alternative to reliable biomedical and industrial resource.

## Figures and Tables

**Table 1:** Comparison of extraction methods and yields for fungal and microalgal chitin.

Source Type	Species	Extraction Method	Yield	Advantages/Limitations	References
Mushroom	*Agaricus bisporus*	NaOH alkaline treatment + ILs/DES + mechanical grinding	7–20.2%	Low mineral content (2.5–7%), sustainable, high yield	[29,31]
Mushroom	*Filamentous Ascomycota*	Alkaline treatment + TEMPO oxidation + Ca^2+^ cross-linking	41.1–49%	High yield, suitable for hemostatic materials	[45]
Yeast	*Pichia pastoris*	Hot alkaline extraction + genetic engineering optimization	2.23 g/L	High yield, enhanced by engineering	[46,47,48]
Other Fungi	*Mucor rouxii*	Alkaline extraction + enzymatic assistance + autolysate replacement	0.135 g/g-AIM	High deacetylation, narrow MW distribution, cost-effective	[48]
Other Fungi	*Fusarium incarnatum*	Alkaline deproteinization + KMnO_4_/oxalic acid + 50% NaOH deacetylation	3.751 g/L	High yield, suitable for scale-up	[49]
Diatom	*Thalassiosira rotula*	Water-based centrifugation + NaCl/EDTA wash + no chemical treatment	Not specified (high AR microrods)	Eco-friendly, no harsh chemicals, fast growth	[15]
Diatom	*Cyclotella cryptica*	Urea/KOH freeze–thaw + ethanol precipitation + H_2_O_2_ depigmentation	272–316 mg/L	High yield, suitable for biomaterials	[50]
Green Microalgae	*Chlorella vulgaris*	Hot NaOH protein removal + acetic/nitric acid purification	0.6%	CO_2_ fixation, no arable land, low yield	[51]

**Table 3:** Functional properties and biomedical applications of fungal and microalgal chitin.

Source Type	Species	Key Physicochemical Properties	Biological Activities	Biomedical Applications	Mechanisms/Advantages	References
Mushroom	*Agaricus bisporus*	Water absorption 674%, self-healing fibers, E = 3415 MPa	Antibacterial, antioxidant, immunomodulatory	Wound healing, hemostasis, packaging	H-bond self-healing, β-glucan enhances binding	[13,37]
Mushroom	*Filamentous Ascomycota*	Water absorption 2400%, strength 175 kPa	Antibacterial, anticancer, anti-inflammatory	Hemostatic dressings, drug delivery	Rapid blood-triggered shape memory, Ca^2+^ promotes clotting	[36,45]
Yeast	*Pichia pastoris*	Shear-thinning, high solubility	Biocompatible, immunomodulatory	Tissue engineering, drug delivery	Genetic engineering, waste utilization	[46,53]
Other Fungi	*Fusarium incarnatum*	High solubility, nanoparticles	Antibacterial, biocompatible, degradable	Wound dressings, drug delivery, environmental remediation	Nano-size enhances contact killing	[49]
Other Fungi	*Ganoderma lucidum*	Gel swelling 1181–1891%, high strength	Antibacterial, antioxidant, hemostatic	Hemostatic gels, tissue engineering	Porous 3D network, platelet adhesion	[5]
Diatom	*Cyclotella* *cryptica*	High solubility, strength ~3 GPa	Antibacterial, hemostatic, biocompatible	Hemostatic dressings, drug delivery	β-type open structure, rapid hydration	[50,65]
Diatom	*Thalassiosira* *rotula*	High aspect ratio, electrorheological	Antibacterial, biocompatible	Biocomposites, green packaging	Hierarchical structure, eco-friendly extraction	[15]
Green Microalgae	*Chlorella vulgaris*	Strong water binding, biodegradable	Antioxidant, biocompatible	Sustainable packaging, wound coatings	Stress-induced layered structure, CO_2_ fixation	[51]

## Data Availability

No new data were created or analyzed in this study. Data sharing is not applicable to this article.
